# Human umbilical vein endothelial cell miRNA secretome highlights endothelial origin of serum miRNAs

**DOI:** 10.1038/s41598-025-10044-8

**Published:** 2025-07-10

**Authors:** Nora J. Doleschall, Zoltán F. Doleschall, Flóra Demeter, Márta L. Debreczeni, Erika Kajdácsi, László Cervenak, Katalin Keltai

**Affiliations:** 1 Institute of Genetics and Cancer, The University of Edinburgh, Western General Hospital Campus, Crewe Road, Edinburgh, EH4 2XU UK; 2 Research Laboratory, Department of Internal Medicine and Haematology, Semmelweis University, Szentkirályi u. 46, Budapest, 1088 Hungary; 3 Department of Pathogenetics, National Institute of Oncology, Ráth György u. 7-9, Budapest, 1122 Hungary; 4 Research Group for Immunology and Haematology, HUN-REN-SU, Szentkirályi u. 46, Budapest, 1088 Hungary

**Keywords:** Molecular medicine, Inhibitory RNA techniques, Microarray analysis

## Abstract

MicroRNAs are key contributors to blood-based biomarker research, however their potential is hindered by the “noise” of their abundance even in healthy blood. Using HUVEC cultures and their conditioned media as a model for endothelium and blood, we were able to detect 574 different microRNAs. 166 of these were exclusively secreted, 155 only intracellular and 253 were found in both states suggesting a highly ordered role in endocrine and paracrine communication. The identified microRNA signatures exhibited higher degrees of variability based on culture conditions rather than genetic background of donors. We found that the endothelial secreted microRNA signature correlates greatly with those found in blood serum (ρ = 0.749 ± 0.044), more so, than leukocyte secretory microRNAs (ρ = 0.531 ± 0.044). These results demonstrate that the endothelium actively secretes microRNAs dominating the microRNA composition of the blood making the endothelial secretory microRNA signatures ideal representation of background “noise” of healthy serum samples. These microRNA signatures are readily adapted to environmental cues; making the standardisation of culture conditions a key concern. Our results also demonstrate that the microarray technology has great use in precision microRNA biomarker discovery using simple models, which should be utilised for further mapping of cell-type specific healthy signatures to further refine blood-based diagnostics.

**Supplementary Information:**

The online version contains supplementary material available at 10.1038/s41598-025-10044-8.

## Introduction

Since their discovery in 1993^1^, microRNAs (miRNAs) have become of interest to many areas of biological research. The molecular function of these ~ 20–25 bp long small non-coding RNAs lies primarily in post-transcriptional regulation of gene expression^2^. They have been found in every conceivable multicellular eukaryote^3–5^ and they play an important role in varied biological processes throughout development^6^ homeostasis and in disease^7^. Generally, miRNAs localise intracellularly in the cytoplasm^8^ however their presence has been observed in bodily fluids as well such as urine, saliva, and blood. Extracellular miRNAs can be found in vesicles, bound to protein complexes or even as cell-free native molecules as well^9^. During early research into miRNA biology, it was thought that extracellular miRNAs are purely a by-product of intracellular metabolism and are therefore discarded into the extracellular space^10^. More recently it is suggested, that miRNAs might very well be actively secreted and have potential roles in cell-cell communication and regulation of gene expression at distant sites^9^.

miRNAs in the bloodstream have been extensively studied in past decades due to their immense potential as biomarkers. They have been shown to have great long-term stability, are expressed in abundance in the blood and have high expression specificity^11,12^. Most studies concerning circulating miRNAs focus on the area of neoplastic disease, nevertheless their functions and utility in the detection of other malignancies has also been suggested^2,12^. When it comes to identification of the origin of circulating miRNAs however, very little is known about physiological homeostatic conditions. Some studies show that the various circulating cellular components of the blood contribute to circulating miRNA populations, yet other potential sources have largely not been investigated^13–17^. Many miRNAs found in the bloodstream have been linked to some organ-specific diseases, such as liver, kidney, and heart malignancies^18–20^ but the miRNA contribution of healthy tissues and organs to the blood still lack discovery.

Identification of baseline values expected in the blood of healthy individuals could greatly improve miRNA biomarker accuracy and for this, mapping the origins of circulating miRNAs is essential. As one of the largest organs in constant contact with the bloodstream, the endothelium has considerable influence on blood composition so we set out to identify whether the endothelium could prove helpful in the establishment of baseline miRNA values in the blood. Human umbilical vein endothelial cells (HUVECs) may be the best model for evaluating the contribution of the endothelium to circulating miRNA. Although HUVECs represent a neonate tissue and endothelium of a specialised blood vessel type, they have two definitive advantages over other available endothelial models. First, they can be utilised in experiments without extensive cell culture, preventing the introduction of substantial in vitro changes in miRNA patterns. Secondly, multiple HUVEC lines from individual donors can be established and cultured using strictly standardised conditions, enabling the observation of distinct genetic background-specific and tissue-specific components of miRNA patterns when compared to several individual blood miRNA profiles.

Many platforms are currently in use for the detection of miRNAs from various sample types, and comparisons between platforms have been extensively discussed elsewhere^21–27^. Here we use miRNA microarray as a gold standard detection method due to its high level of reproducibility, great dynamic range, and specificity. Application of microarrays on in vitro models of the vasculature and blood as well as blood-serum and plasma samples allows us to report here the contribution of the endothelium to circulating miRNA species, providing an important step towards precision diagnostics.

## Results

### Establishing endothelial miRNA signatures

**Fig. 1:**
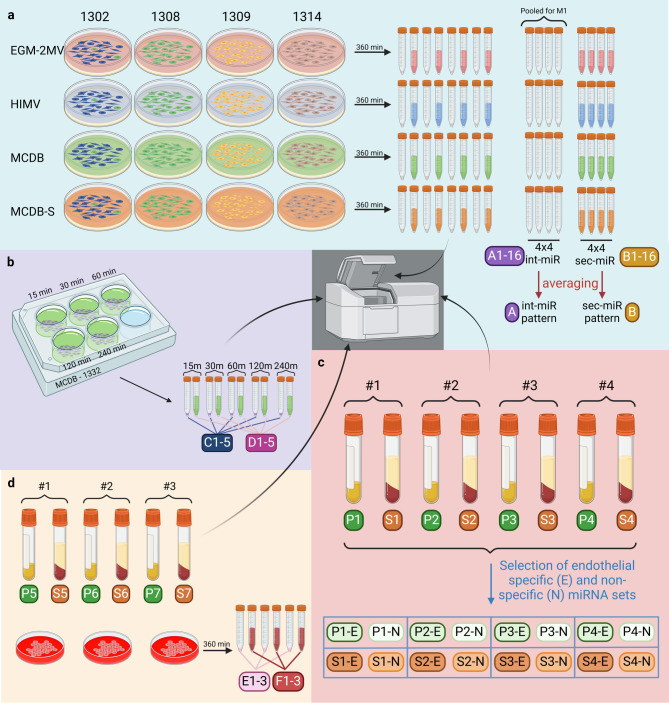
Experimental setup and sample directory. Overview of all samples generated throughout the study, measured on Agilent microarray platform. (**a**) HUVEC lines were obtained from 4 donors (1302–1314) and were cultured in 4 different cell culture media (EGM-2MV; HIMV; MCDB and MCDB-S) Samples were generated from conditioned media (B1-16) and centrifuged cell pellets (A1-16). (**b**) HUVEC line from donor 1332 was cultured in MCDB medium for 15 to 240 min in triplicates, then samples generated from cell pellets (C1-5) and conditioned media (D1-5). (**c**) Blood drawn from 4 healthy donors (#1–4) was collected as plasma (P1-4) and serum (S1-4). Resulting miRNA expression values were categorised based on presence (SE/PE) or absence (SN/PN) in samples from (A). (**d**) Blood was drawn again from healthy donors (#1–3) as plasma (P5-7) and serum (S5-7) and blood cell cultures were established as well from the same. Samples were generated from blood cell pellets (E1-3) and their conditioned media (F1-3).

To model the contribution of the endothelium to the miRNA expression signature of blood samples we studied the miRNA expressions of four different primary HUVEC cultures derived from healthy individuals. As the equivalent of the bloodstream in this model, the expression pattern of the conditioned media from these cultures was also measured. Previous studies indicate, that these primary cell lines exhibit differential behaviour based on the culture conditions^28,29^. Aiming to establish average endothelial miRNA signatures we wanted to determine whether different cell culture media could introduce additional variability to the gene expression patterns generated. Therefore, all four HUVEC cultures were maintained simultaneously in four different commonly used cell culture media yielding sixteen samples (Fig. 1a, A1-16).

**Fig. 2:**
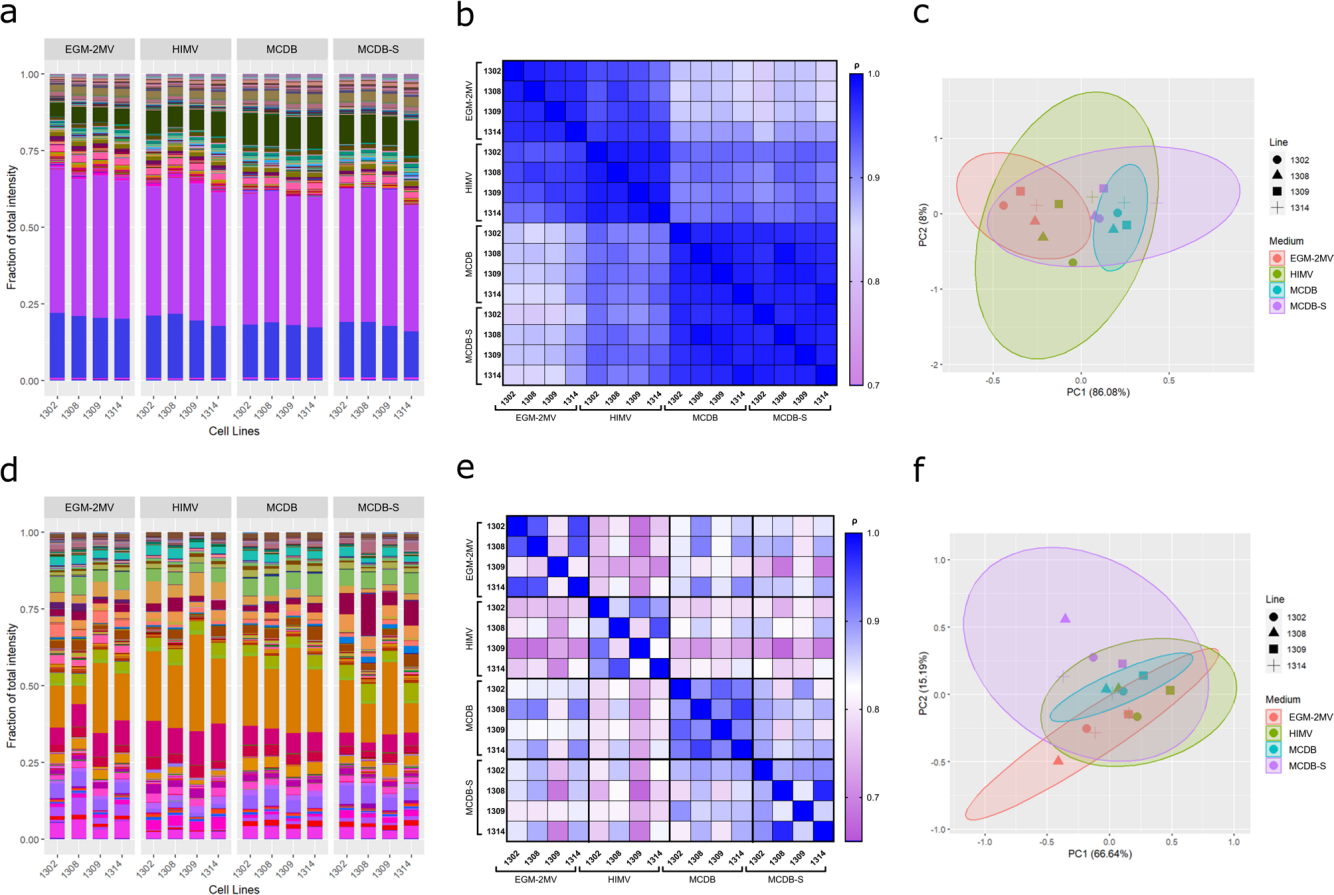
Culture conditions modify intracellular and secretory miRNA signatures in HUVEC cultures. Visualisation of (**a**) int-miR and (**d**) sec-miR signature of HUVEC lines (1302, 1308, 1309 and 1314) cultured in four different media. Correlation matrix showing Spearman coefficients between the sixteen cell line-culture condition combinations for (**b**) int-miRs and (**e**) sec-miRs. Clustering based on principal components shows a slight dependency on culture media on (**c**) int-miR signatures, which is not observable in the case of (**f**) sec-miR signatures.

The sixteen cultures expressed on average 398(± 21) miRNAs (Supplementary Figure S1). The comparison of the patterns of intracellular miRNAs (int-miRs) in these samples yielded high degrees of correlation overall (ρ = 0.928 ± 0.047, Fig. 2a-b, Supplementary Table S1), and clustering based on cell culture conditions was observed (Fig. 2c). Correlation between miRNA expression patterns of a single cell line across different conditions was ρ = 0.924 (± 0.047) which was significantly lower (*p* = 0.0001), than correlations measured across different cell lines cultured in the same medium (ρ = 0.977 ± 0.005) (Supplementary Figure S1). This result suggests the need for standardised culture conditions when looking at miRNA expression signatures.

**Fig. 3:**
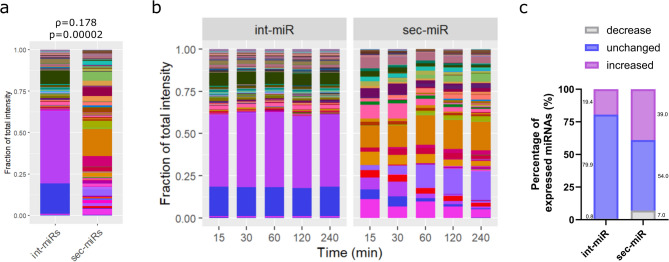
The endothelium produces vastly different miRNA signature for intracellular use and for secretion. (**a**) Visualisation and Spearman correlation of average int-miR (*n* = 16) and sec-miR (*n* = 16) signatures of HUVEC lines. P value shows confidence in Spearman coefficient. (**b**) Visualisation of int-miR and sec-miR patterns of a HUVEC culture over four hours (**c**) Changes in int-miR and secmiR expressions over time. miRNAs were assigned to each category based on Pearson correlation of normalised expression with time, *p* < 0.05.

An average endothelial int-miR profile was generated from the sixteen HUVEC samples (Fig. 1a, A), which gives us an estimate of miRNA signatures expected to show up in the bloodstream upon vascular injury and endothelial apoptosis. However, many organs and tissues have been observed to release cell-free miRNAs into the bloodstream, and we hypothesised the endothelium to be no different. To establish the signature of endothelial secretory miRNAs (sec-miRs), RNA was extracted from the conditioned media of the sixteen cultures and measured as before (Fig. 1a, B1-16). On average 210(± 73) miRNAs were detected in the conditioned media of the sixteen cultures (Supplementary Figure S1). A higher degree of variation in expression profiles was observed between these sixteen replicates compared to variation between int-miR patterns, however the correlation between the samples was still high (ρ = 0.825 ± 0.067, Fig. 2de, Supplementary Table S2). The effect of different culture conditions was still observed in the sec-miR signatures (*p* = 0.001 compared to the effect of genetic background), however the clustering was not as obvious as with int-miR signatures (Fig. 2f, Supplementary Figure S1). Interestingly, the average sec-miR and int-miR signatures (Fig. 1a, A and B) had very low correlation with each other (ρ = 0.178, *p* = 0.00002 Fig. 3a). Out of all 574 miRNAs detected across the sixteen cell and conditioned media samples, 166 were found only to be secreted, 155 to be exclusively intracellular and 253 expressed both intra- and extracellularly (Supplementary Figure S1, Supplementary Table S3). MiRNAs that were found in both the cellular fraction and the conditioned media are expressed in vastly different proportions in the two sample sets, so that their correlation is not significant (ρ = 0.091, *p* = 0.1479), suggesting the presence of these miRNAs in the medium is also not the product of necrotic cells bursting their intracellular contents into the medium. This result supports the notion, that the endothelium actively produces miRNAs for secretion, rather than simply releasing or disposing of them as by-products of regular cellular function.

Sequential analysis of sec-miRs from a HUVEC culture in MCDB medium between 15 min and 4 h from seeding in low-serum conditions shows a steady increase in total signal intensity. The int-miR signal intensity however does not seem to consistently increase with time, suggesting that the cells are actively producing miRNAs for secretion while maintaining internal miRNA levels (Fig. 1b, Supplementary Figure S2). Almost half (46) of all detected sec-miRs from the time-course (total 100) show significant change in their normalised expression in correlation with time. Most of these (39/46) are upregulated while a small subset (7/46) decreases from initial high proportions (Fig. 3b-c, Supplementary Table S4). These secmiR changes occur while the pattern of intmiRs largely remain unchanged (304/381, no significant correlation with time), suggesting that the sec-miR content is actively adapted over time (Fig. 3b-c). Observing more closely the synchronous changes occurring intra- and extracellularly, the 7 sec-miRs found to decrease over time are all significantly increased intracellularly, implying the presence of intracellular reserves being secreted soon after plating (Supplementary Table S4). Looking at the intensity changes rather than normalised proportional changes, we can see significant increase in the expression of 6 out of the above-mentioned 7 sec-miRs in their secretory capacity implying that their secretion is slower than that of other miRNAs suggesting their functional importance at the early stages of environmental changes.

### Endothelial sec-miR signature dominates in blood serum miRNA expression patterns

**Fig. 4:**
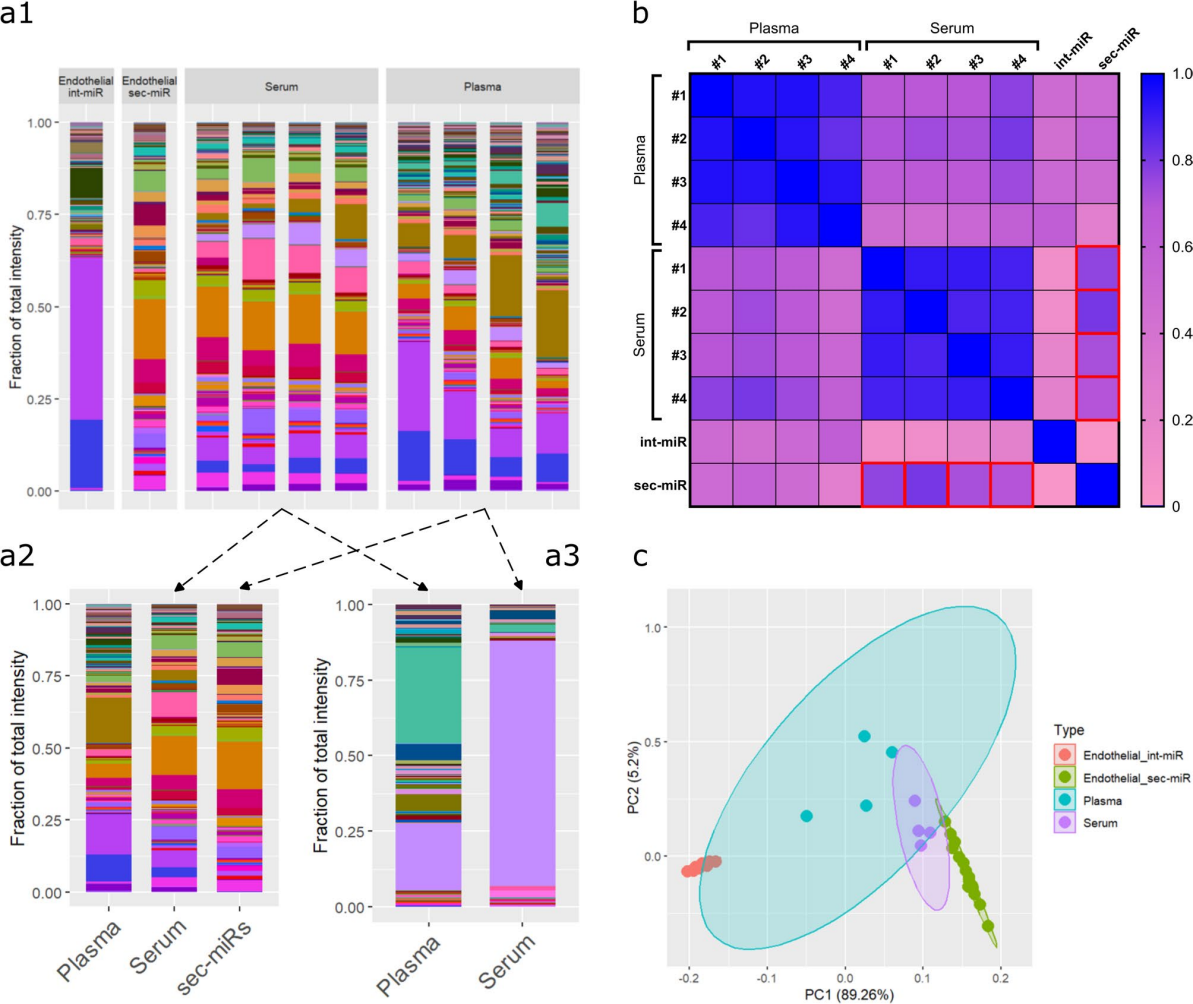
Endothelial sec-miR signature is dominant in blood serum but not in plasma samples. (**a**) Visualisation of miRNA signature of four paired plasma and serum samples along with endothelial int-miR and sec-miR signatures. (**a1**) shows overall serum and plasma patterns, (**a2**) highlights endothelial and (**a3**) highlights non-endothelial miRNAs in blood samples (*n* = 4) (**b**) Correlation matrix of Spearman coefficients calculated between blood samples and endothelial miRNA signatures (*n* = 16) considering endothelial miRNAs only. **(c)** Clustering of four paired blood samples and endothelial int-miR and sec-miR samples.

The amount and intensity of miRNAs produced by the endothelium both for secretion and internal use, highlights the need to use reference signatures to reduce the noise of healthy homeostasis when analysing blood sample miRNA expressions. Therefore, we determined the miRNA signatures of four paired plasma and serum samples from healthy individuals to identify the importance of endothelial miRNA patterns in these (Figs. 1c and 4a1-a3). On average we were able to detect 406(± 59) and 216(± 46) miRNAs in the plasma and serum samples respectively (Supplementary Figure S3). The endothelial int-miR and sec-miR signatures previously identified (Fig. 1a, A and B) were compared to the signatures of the blood plasma and serum samples (Fig. 1c, P1E-1E and S1E-1E). Interestingly, the endothelial sec-miR signature which is used as the proxy for blood in our experimental model, correlates significantly better (*p* = 0.029) to serum samples (ρ = 0.749 ± 0.044) than plasma samples (ρ = 0.450 ± 0.131) observable both by Spearman correlation (Fig. 4b, red box, Supplementary Figure S3) and principal component analysis (Fig. 4c) results (Supplementary Table S5).

The signature of miRNAs with possible endothelial origin in serum and plasma samples do not correlate very strongly (ρ = 0.727 ± 0.025) considering that these are paired samples from the same individuals. So, to address the possibility that this is an artefact of excluding miRNAs of non-endothelial origin from the above analysis, Spearman correlation coefficients were calculated for each paired serum and plasma samples (Fig. 1c, P1-4 and S1-4). This analysis produced similar results to the above (ρ = 0.660 ± 0.045, Fig. 4a1). To assess whether the differences between sample types could be attributed to endothelial function we further analysed the correlation of miRNAs of nonendothelial origin between paired samples separately (Fig. 4a3, Fig. 1c, P1N-4N and S1N-4N). This analysis yielded even lower coefficients (ρ = 0.309 ± 0.047), confirming that the variation is not only present in endothelium-related miRNAs.

**Fig. 5:**
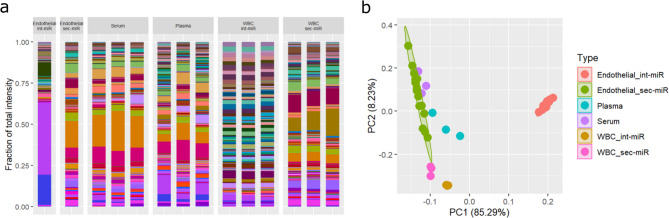
Leukocytes contribute less to blood serum and plasma miRNA signatures than the endothelium. (**a**) Visualisation of overall miRNA patterns of three paired samples of blood serum, blood plasma, int-miR and sec-miR signatures from cultured blood cells along with endothelial int-miR and sec-miR signatures with (**b**) showing clustering of same.

Seeing as the serum and plasma samples contained additional miRNAs not expressed in our endothelial baseline (15 ± 9 and 50 ± 15 respectively), we used our experimental setup described for endothelial cell culture in vitro to identify the contributions of the cellular components of the blood to these samples. Signatures of int-miR and sec-miR expression were established for isolated generic blood cell cultures and conditioned media from three healthy donors as well as miRNA signatures for three matched serum and plasma samples from the same donors (Fig. 1d). We detected 1035(± 157) miRNAs in white blood cell (WBC) cultures and 795(± 40) in WBC conditioned media. Number of detected miRNAs between the first and second set of plasma and serum measurements was not changed significantly (*p* = 0.21 and *p* = 0.53 respectively Fig. 1c and d). Including the WBC samples in clustering analyses showed that the cellular components of the blood likely contribute less to the overall miRNA pattern of a healthy blood sample compared to the contribution of the endothelium (Fig. 5). This result highlights the advantage of using endothelial expression as baseline in blood serum measurements.

## Discussion

In this study we set out to provide a baseline miRNA signature to be used as background for bloodbased miRNA studies and diagnostics. Using a model of HUVEC cultures and their conditioned media as a proxy for the vasculature and blood respectively, we were able to show that the endothelium secretes miRNAs destined for extracellular use from the start and that this signature is adapted over time. Furthermore, we provide evidence that these endothelial sec-miR signatures strongly contribute to the miRNA content of blood serum samples, even more so than the cellular components of the blood, making them ideal candidates for healthy baseline establishment.

Previous studies have reported the various ways, that the endothelium and endothelial cultures respond to external stimuli, highlighting the importance of the standardisation of culture conditions^28,29^. Similarly, we show here that the influence of culture media on the miRNA patterns of HUVEC cultures is more profound than the differences caused by inherent genetic differences between samples (Fig. 2a-c.). Consideration should therefore be given to the identification of adequate culture conditions, when conducting transcriptomic studies of in vitro endothelial models.

The presence and relevance of secretory miRNAs has been long discussed and reviewed since their first discovery^30^ first hypothesised to be no more than by-products of cellular activity^10^. More recently it is believed that miRNA secretion is a purposeful and regulated process^9,31,32^ which we corroborate by showing that the endothelium produces a panel of miRNAs only for secretion, and that the normalised expression signatures also follow vastly different patterns between intracellular int-miRs and secretory sec-miRs (Supplementary Figure S1 and Fig. 3a). The endothelium has been reported previously to secrete and take up circulating miRNAs in various processes and diseases^33–37^ however only a handful of these have been identified so far and were tested for functional relevance. Here we provide a panel of over 400 miRNAs (Supplementary Figure S1) shown to be secreted by the endothelium, 29 of which have never been described in literature in any context whatsoever, alongside additional 3 novel miRNAs found to be endothelial int-miRs with no literature references at the time of preparation of this manuscript (Supplementary Table S3). These results highlight the need to utilise multi-marker miRNA panels in a context dependent manner, as they may mark entirely separate processes depending on their relative abundance and passive or active origin in the blood stream. As an example, the here described endothelial int-miR panels might be used as markers of vascular damage, whereas the sec-miR signatures identified, will likely better be utilised as tools to reduce noise and background of healthy blood homeostasis in liquid biopsies.

Beside the fact that miRNAs are secreted with purpose we report for the first time their signatures to be actively adapted over time, as rapid and sustained response to changes in environment (Fig. 3b-c). Our results suggest that endothelial sec-miRs are more relevant in cell-cell communication and maintenance of the homeostatic environment, while int-miRs might have a more conservative role with less potential to change and adapt. Previously described miRNA reserves^8^ secreted early after placing cell cultures into a new environment might serve as signals to the environment announcing cell identity followed by adaptation to feedback signalling or lack thereof, shaping the external environment and buffering environmental contingencies^38^. Previous research has introduced miRNAs as intercellular signalling molecules especially in the context of cancer^39–41^ and our findings further suggest their roles not simply as messengers and intercellular gene expression modulators but also as regulators of homeostasis.

Most research aiming to identify the origin of circulating miRNAs, focuses on disease and especially tumour derived miRNA populations. Even when considering non-tumour miRNAs, many studies attribute their presence to blood cells, especially leukocytes, linking their roles to immunogenic processes^13–15,42−44^. Here we demonstrate, that the endothelium is a great contributor to circulating miRNAs detectable in plasma and especially serum samples even in healthy individuals (Fig. 4). The contribution of the endothelium proved to be even more influential than that of the cellular components of the blood (Fig. 5b), suggesting perhaps the role of the endothelium in establishing the homeostatic conditions in the blood is more pronounced, than the role of circulating leukocytes, on which a lot of studies place their focus. We also show that miRNA patterns greatly differ between blood plasma and serum samples (Fig. 4a), emphasizing the importance of standardised sample collection and study comparability. Differences between plasma and serum samples can potentially be attributed to thrombocyte function or haemolysis occurring during sample collection^45^. Nonetheless, determining baseline miRNA signatures will have to be sample type specific in the future. Our results comparing the contribution of the endothelium and the cellular components of the blood to plasma and serum miRNA signatures also highlight the complex interplay of organs, tissues, and cell types in contact with the blood stream all asserting influence on its miRNA content in various sample types.

While our findings provide valuable and promising insights, the study has some inherent limitations we ought to acknowledge. Throughout our experiments we used HUVECs as our endothelial cell model, despite the well-known diversity across endothelial tissue- and vessel types even at translatome level^46,47^. However we find justification for using HUVECs in this in vitro setting, as Cleuren and colleagues reported that heterogeneous in vivo endothelial cell transcriptomes and translatomes, exhibit a strong drift towards a common endothelial cell phenotype following isolation and in vitro expansion regardless of organ of origin. Making sure that sec-miRs are obtained only after a short period of in vitro expansion and from cultures with strictly uniform culture conditions was most efficiently achieved using HUVECs. With opting for this model, we initially expected to find low or moderate correlation between HUVEC sec-miRs to blood serum miRNA patterns, as HUVECs represent the endothelium of a single, foetal type of blood vessel, with some marked differences to adult endothelium. Surprisingly, the correlation found was striking, leading us to postulate that contributions of other endothelial cell types may yield even higher degrees of correlation, this however requires extensive further validation.

Overall, this study provides significant insights into the pronounced contribution of the endothelium to the miRNA contents of the blood, opening new avenues of investigation to identify the contribution of other key players in shaping the miRNA signatures of blood samples such as leukocytes, the liver and many more. This study provides a proof of concept and contributes the basis for further largescale research into the intricacies of sec-miRs in the blood to aid with miRNA-based precision diagnostic approaches.

## Methods

### Blood samples

Blood samples were taken via venepuncture, and were immediately transferred to the processing laboratory, where the cells and the supernatant – serum and EDTA-anticoagulated plasma – were separated by centrifugation. Serum and plasma aliquots were prepared, immediately frozen and stored at − 80 °C until the measurements were performed.

### Cell cultures

Endothelial cells were prepared by collagenase digestion from fresh umbilical cords obtained during normal deliveries of healthy neonates as described earlier^48,49^. HUVECs were cultured in gelatine pre-coated flasks (Corning, NY, USA) in MCDB-131 medium (ThermoFisher Scientific, Waltham, MA, USA) completed with 5% filtered, heat inactivated bovine serum (PAN Biotech), 1 ng/mL human recombinant basic fibroblast growth factor (bFGF), 2 ng/mL human recombinant epidermal growth factor (EGF, R&D Systems), 0.3% insulin–transferrin–selenium (ITS, ThermoFisher Scientific), 1% chemically defined lipid concentrate (ThermoFisher Scientific), 1% Glutamax solution (ThermoFisher Scientific), 1% penicillin–streptomycin antibiotic solution, 5 µg/mL ascorbic acid, 250 nM hydrocortisone, 10 nM HEPES, and 7.5 U/mL heparin; hereinafter referred to as MCDB. HUVECs were used between passage 2 to 4.

In specific experiments, cells were detached by trypsin-EDTA then seeded onto plates in four different types of media. Besides MCDB, its reduced growth factor containing variant, MCDB-S (1% FBS, no recombinant EGF, bFGF, nor ITS), HIM-V medium (AIM-V from ThermoFisher completed with 1% filtrated, heat inactivated FBS, 1 ng/mL bFGF, 2 ng/mL EGF and 7.5 U/mL heparin), and EGM-2MV (Lonza, CA, USA) were utilized. After incubation, supernatants were collected, cell debris was removed by centrifugation at 5000 g, and the samples were stored at – 80 °C until RNA extraction. Attached cells were washed twice with HBSS buffer then 500 µL of TRI Reagent (Merck-Sigma) was added to each well. Cell lysates were also stored at – 80 °C until RNA extraction. RNA extraction was also performed from blank media used as background. (Supplementary Figure S4)

White blood cells (WBC) were collected from EDTA-anticoagulated whole blood after removing the plasma by centrifugation. WBCs were washed twice with PBS then incubated in RPMI-1640 without FBS for 6 h. WBCs as well as supernatants were stored at – 80 °C until RNA extraction.

### RNA extraction and microarray data acquisition

Small RNA enriched total RNA extraction from all samples was done using miRNeasy kit (Qiagen, 217004). For fresh cell culture media, cell culture serum, conditioned media and blood serum and plasma samples 2 × 200ul was used for extraction on the same column for appropriate RNA quantities.

RNA input and labelling parameters were used according to manufacturer recommendations (Agilent). 100 ng total RNA was used for dephosphorylation and endlabelling reactions with Cy3-pCp, purified on Micro Bio-S P-6 gel columns (Bio-Rad, 732–6221), dried and hybridised over 20 h at 55 °C rotating at 20 rpm using Agilent hybridisation oven (G2545A, Agilent Protocol Version 3.1.1*)*.

Hybridised slides were scanned using Agilent Microarray Scanner (G2505C, Agilent Scan Control (A.8.4.1)). Feature recognition and spot alignment were done using Agilent Feature Extraction software (10.7.3.1).

### Evaluation of microarray quantitative capabilities

MiRNA expression in samples was measured on the Agilent Microarray platform. To ascertain that the probe-set used for our miRNA analyses is adequate for quantitative measurements, a serial dilution of pooled RNA isolates from the sixteen HUVEC samples was applied to the following steps of the microarray protocol (Fig. 1a, M1). The serial dilution included the protocol standard of 100 ng input RNA, and was expanded to include both relatively small and high values (12.5 ng-150 ng), to show to what limits our system and our normalisation method could tolerate variable inputs. Pearson correlation coefficients were calculated for each probe across intensity values and input concentrations and probes that did not reach significant correlation (2.7% of probes) were filtered out and disregarded in further analyses (Supplementary Figure S5). An intensity threshold was also calculated from the average intensity of miRNAs, which were newly detected between pooled samples with each increase of input material (1.36).

Further to validating the quantitative capabilities of this setup, miRNA intensity values in the pooled samples were normalised as a fraction of total intensity of each sample and compared to each other as whole datasets using Spearman correlation (Supplementary Figure S5). Comparing the normalised pattern of miRNAs expressed in the different pooled samples showed high correlation especially with RNA inputs of 50 ng or higher. The visible shift in expression patterns across the six samples (Supplementary Figure S5) is due to the differences in slope of the different probes shown on Supplementary Figure S5, however even beside these differences, the correlation of the patterns is still high. This high correlation is observed despite the great differences in total intensity values between the samples (Supplementary Figure S5), depicting the advantage of utilising the fraction of total intensity values for each probe as normalisation for comparative approaches across samples, even in the absence of internal and external standards, independent of the potential variability in the amount of input RNA, up to 50% both directions (Supplementary Figure S5, red boxes).

### Data analysis and graphics

Data analyses were carried out using various softwares and packages. Normalisation was carried out in R (version 4.2.2 https://cran.r-project.org/bin/windows/base/old/4.2.2) in Rstudio (build 2022.12.0 + 353.pro20) using total intensity values of each sample. Pearson and Spearman correlation coefficients (ρ value) were calculated using GraphPad Prism (version 9.4.0, http://www.graphpad.com) along with Student’s t-tests, Welch’s t-tests and MannWhitney tests. When correlation matrices were calculated, ρ**-**values are reported as the mean of all pairwise correlations in the relevant fields of the matrix and the standard deviation from this mean. P values below 0.05 were considered significant. Principal component analyses were carried out using *prcomp* function from base R package *stats*. P values for correlation matrices are shown in Supplementary tables, whereas p values for t-tests and similar comparisons are shown on figures or in the text. Sample pattern bar plots were created using R package *ggplot2* along with PCA plots. Colour codes for each miRNA is used consistently across all figures and is listed in Supplementary Table S6. Figure 1 was created with BioRender (https://www.biorender.com/).

## Electronic supplementary material

Below is the link to the electronic supplementary material.

Supplementary Material 1

## Data Availability

The data described in this article are openly available on ArrayExpress (https://www.ebi.ac.uk/biostudies/ArrayExpress/studies) under Accession numbers: E MTAB-14672; E-MTAB-; E-MTAB-14717 and E-MTAB-14718.
