# Supplementary material for: Human umbilical vein endothelial cell miRNA secretome highlights endothelial origin of serum miRNAs
Source: Sci Rep. 2025 Jul 10;15:24960. doi: 10.1038/s41598-025-10044-8 (PMC12246433; doi:10.1038/s41598-025-10044-8)
Supplement: Supplementary file 1 — Supplementary Material 1 [file 41598_2025_10044_MOESM1_ESM.pdf]

Human umbilical vein endothelial cell miRNA secretome highlights endothelial origin of serum miRNAs

Nora J. Doleschall[1], Zoltán F. Doleschall[2,3], Flóra Demeter[2], Márta L. Debreczeni[2], Erika Kajdácsi[2,4], László Cervenak[2,*], Katalin Keltai[2]

[1]Institute of Genetics and Cancer, The University of Edinburgh, Western General Hospital Campus, Crewe Road, Edinburgh, EH4 2XU

[2]Research Laboratory, Department of Internal Medicine and Haematology, Semmelweis University, Szentkirályi u. 46., Budapest 1088, Hungary.

[3]Department of Pathogenetics, National Institute of Oncology, Ráth György u. 7-9., Budapest 1122, Hungary

[4]Research Group for Immunology and Haematology, HUN-REN-SU, Szentkirályi u. 46., Budapest 1088, Hungary

[*] Corresponding author: cervenak.laszlo@semmelweis.hu

Supplementary Figure S1. (a) Number of miRNAs detected in each of four HUVEC cell lines cultured in four types of cell culture media (Paired student's t-test). Correlations between miRNA patterns of the same grouped based on comparison constants. Correlations were measured for both intracellular **(b)** and secreted **(c)** miRNA signatures (Unpaired student's t-test). **(d)** Outline of samples classified into groups for comparisons shown in **(b)** and **(c)**.**(e)** Number of miRNAs found in the cellular fraction (int-miR) or conditioned medium (sec-miR) of the 16 cell cultures. Error bars show standard deviation.

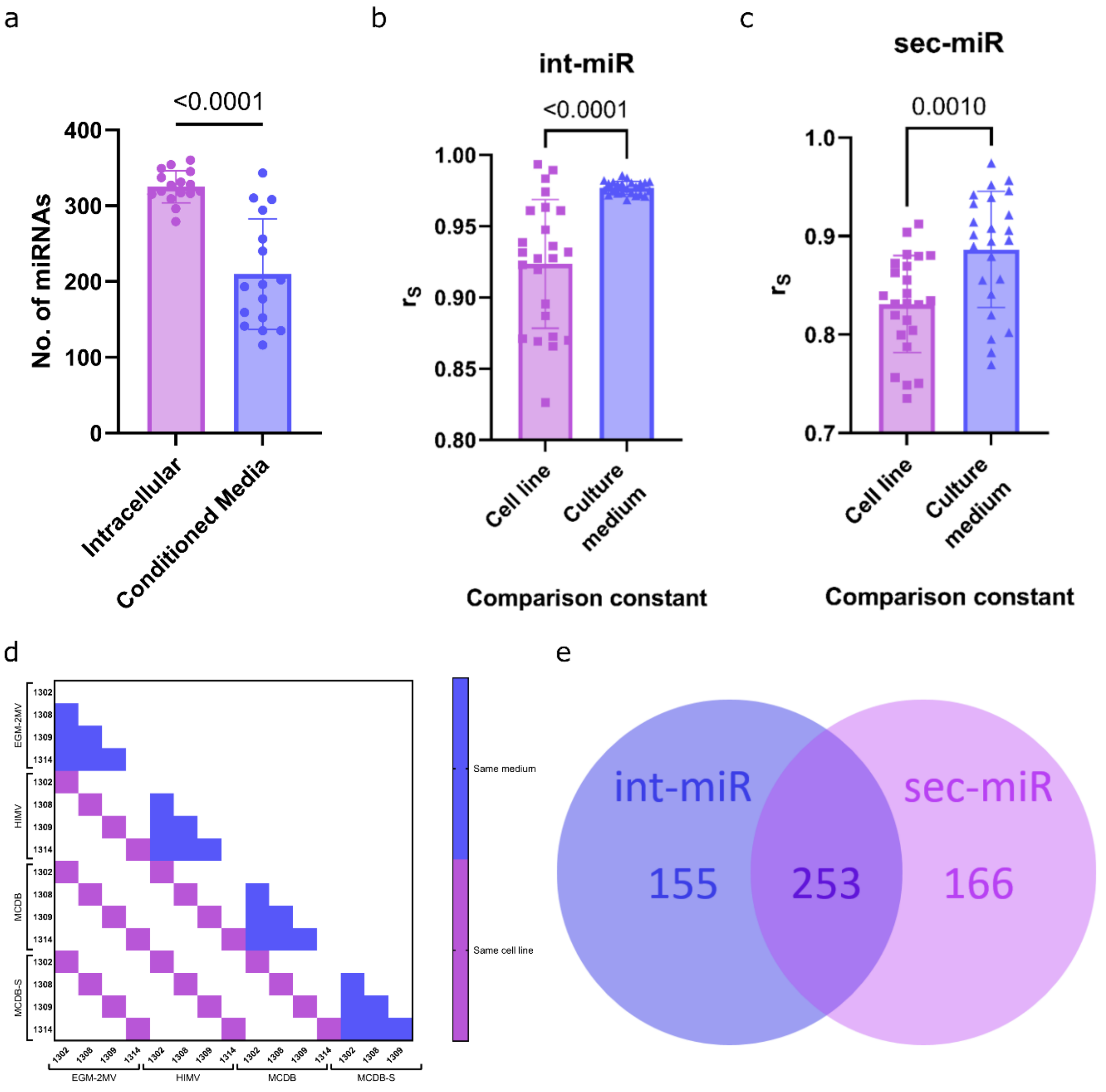

Supplementary Figure S2. Total intensity of miRNAs detected over time in cell pellets and conditioned media. Error bars show standard deviation.

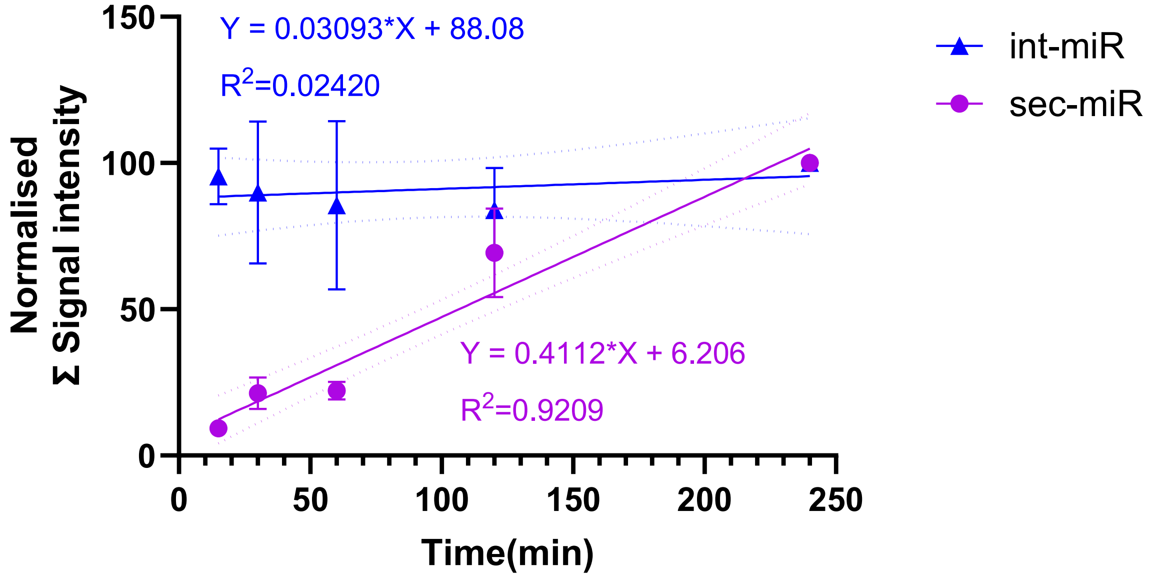

Supplementary Figure S3. (a) Number of miRNAs detected in the first four paired plasma and serum samples (Paired Student's t-test). **(b)** Total signal intensity of the same (Paired Student's t-test). **(c)** Spearman correlations of paired individual plasma and serum samples to the average sec-miR expression pattern of HUVEC cultures (Paired Student's t-test). Error bars show standard deviation.

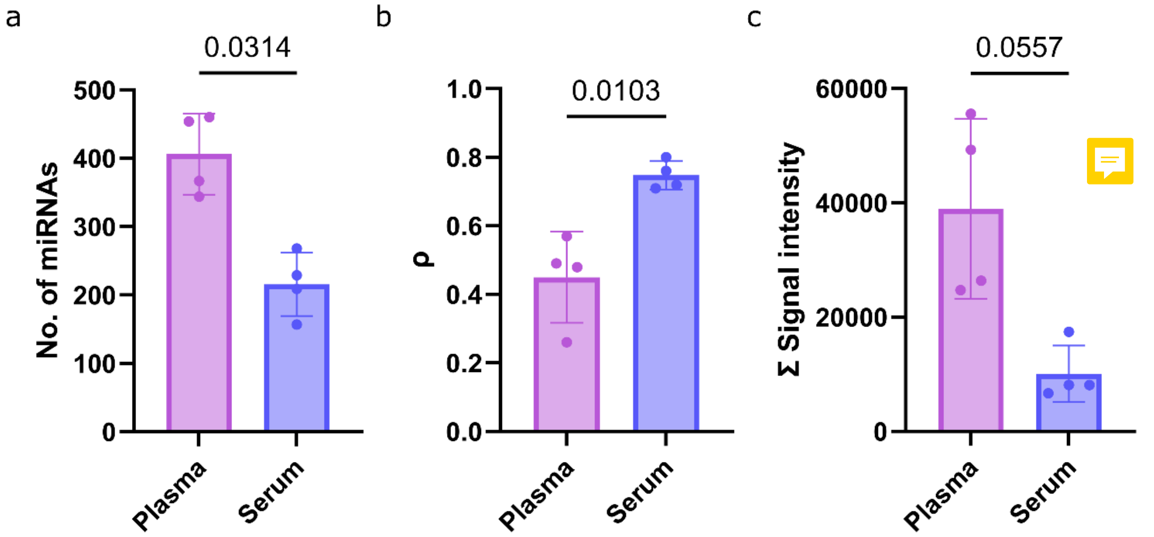

Supplementary Figure S4. Total intensity of blank cell culture media used to eliminate the interpretation of detected cross-species and non-specific hybridisation events as real signal. Intensity values for each miRNA were subtracted from all sec-miR intensities. Error bars show standard deviation.

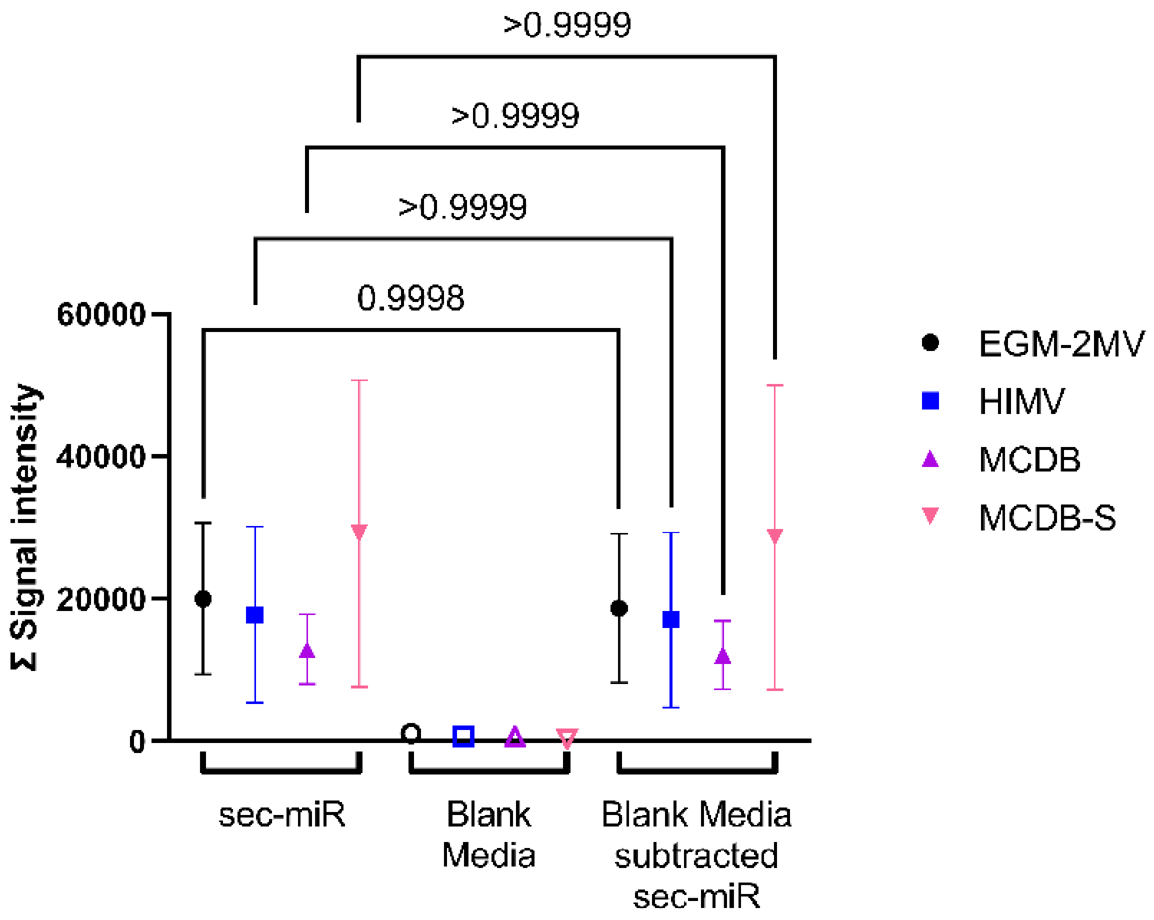

Supplementary Figure S5. (a) Red symbols and lines show the miRNA probes filtered out from further analyses based on the significance of correlation with input RNA concentration ($p<0.05$). **(b)** Visualisation of normalised miRNA expression signatures in sixteen pooled samples based on varying input material. **(c)** Spearman correlation coefficients of miRNA expression patterns of pooled samples across a range of input material were calculated to assess reliable input range, marked by red boxes around the protocol standard of 100ng. **(d)** Total intensity values of serial dilution of input RNA from sixteen pooled samples.

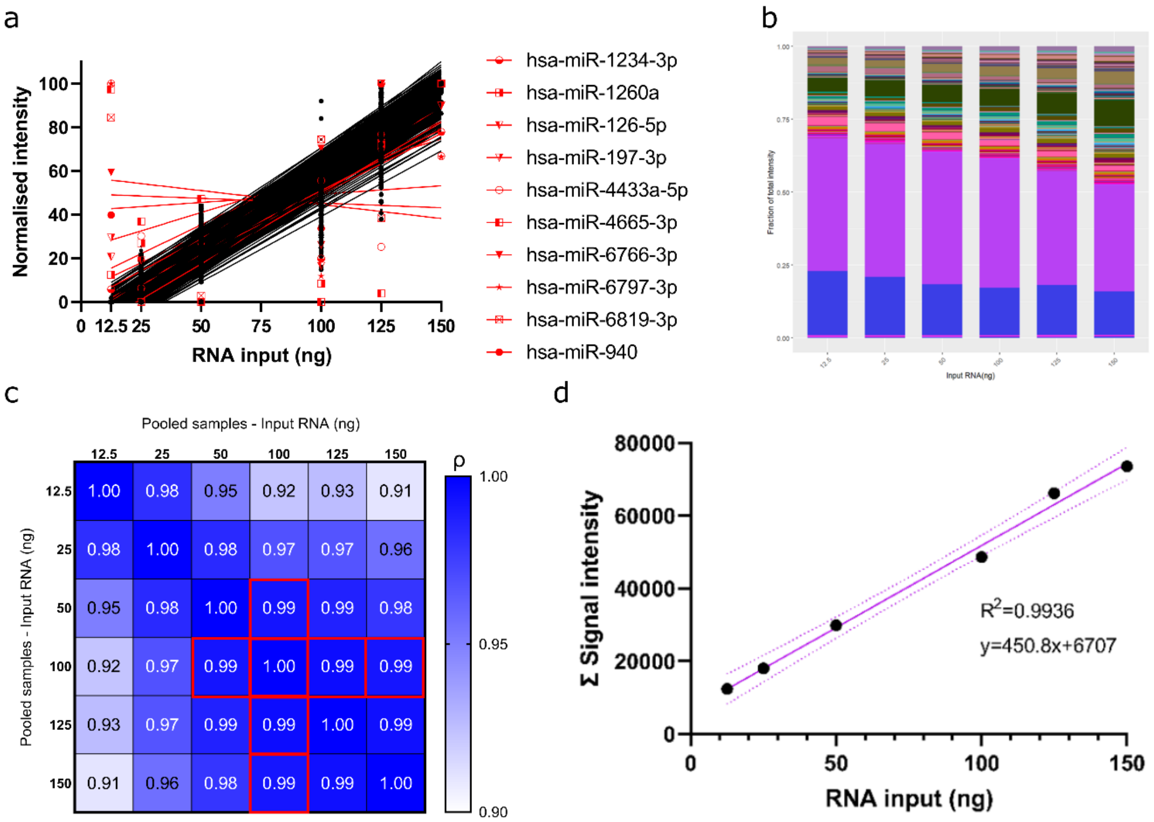

Supplementary Table S1. Spearman correlation coefficients and p values of intracellular miRNA expression signatures of four HUVEC cell lines cultured in four different media. Bold and Italic values are used for calculations in ~~Extended~~ Supplementary Data Figure 1b.

r_s		EGM-2MV				HIMV				MCDB				MCDB-S			
		1302	1308	1309	1314	1302	1308	1309	1314	1302	1308	1309	1314	1302	1308	1309	1314
EGM-2MV	1302	1															
	1308	*0.97987*	1														
	1309	*0.97105*	*0.9733399*	1													
	1314	*0.96845*	*0.9717938*	*0.9734436*	1												
HIMV	1302	**0.947592**	0.9396765	0.9426571	0.9568056	1											
	1308	0.950566	**0.9632223**	0.9558012	0.9628625	*0.9821209*	1										
	1309	0.944756	0.945037	**0.9608778**	0.960656	*0.980916*	*0.9806246*	1									
	1314	0.92388	0.9237343	0.9410571	**0.9609754**	*0.9812708*	*0.9732448*	*0.983885*	1								
MCDB	1302	**0.865805**	0.8458374	0.8594246	0.8824935	**0.9318461**	0.9110134	0.9075708	0.9187835	1							
	1308	0.864647	**0.869252**	0.8619751	0.8891395	0.9229283	**0.9274213**	0.9094429	0.9169848	*0.9750248*	1						
	1309	0.862414	0.8606045	**0.8710649**	0.8935032	0.9298831	0.9235395	**0.919577**	0.9265849	*0.9779315*	*0.9855282*	1					
	1314	0.84809	0.845297	0.8550267	**0.8953853**	0.9271218	0.9148384	0.911105	**0.9360169**	*0.9724471*	*0.975202*	*0.9793983*	1				
MCDB-S	1302	**0.826266**	0.8191469	0.8323578	0.8634109	**0.9229338**	0.9044243	0.898474	0.9192253	**0.9740314**	0.96761	0.9721598	0.9757725	1			
	1308	0.86413	**0.8699486**	0.864781	0.8902746	0.9264906	**0.9316277**	0.9151462	0.9213521	0.9717115	**0.9934558**	0.9836311	0.9771645	*0.9714598*	1		
	1309	0.864039	0.8647723	**0.87265**	0.8944346	0.9360145	0.9297165	**0.927665**	0.9341496	0.9675027	0.9751348	**0.9834818**	0.9746363	*0.9795318*	*0.9799809*	1	
	1314	0.837992	0.8384794	0.8493496	**0.887025**	0.9293657	0.9141781	0.9137252	**0.9385197**	0.9649498	0.9696445	0.9731202	**0.9893892**	*0.9803498*	*0.9748313*	*0.9766494*	1

p		EGM-2MV				HIMV				MCDB				MCDB-S			
		1302	1308	1309	1314	1302	1308	1309	1314	1302	1308	1309	1314	1302	1308	1309	1314
EGM-2MV	1302		Approximate	Approximate	Approximate	Approximate	Approximate	Approximate	Approximate	Approximate	Approximate	Approximate	Approximate	Approximate	Approximate	Approximate	Approximate
	1308	1.7E-264		Approximate	Approximate	Approximate	Approximate	Approximate	Approximate	Approximate	Approximate	Approximate	Approximate	Approximate	Approximate	Approximate	Approximate
	1309	2.9E-235	6.7E-242		Approximate	Approximate	Approximate	Approximate	Approximate	Approximate	Approximate	Approximate	Approximate	Approximate	Approximate	Approximate	Approximate
	1314	2.2E-228	2.25E-237	3.26E-242		Approximate	Approximate	Approximate	Approximate	Approximate	Approximate	Approximate	Approximate	Approximate	Approximate	Approximate	Approximate
HIMV	1302	6.4E-188	8.55E-177	8.5E-181	2.76E-203		Approximate	Approximate	Approximate	Approximate	Approximate	Approximate	Approximate	Approximate	Approximate	Approximate	Approximate
	1308	1.5E-192	4.07E-216	1.87E-201	2.44E-215	4.46E-274		Approximate	Approximate	Approximate	Approximate	Approximate	Approximate	Approximate	Approximate	Approximate	Approximate
	1309	9.5E-184	3.77E-184	3.51E-211	9.93E-211	8.14E-269	1.36E-267		Approximate	Approximate	Approximate	Approximate	Approximate	Approximate	Approximate	Approximate	Approximate
	1314	1.6E-158	2.31E-158	1.27E-178	2.22E-211	2.49E-270	1.29E-241	1.83E-282		Approximate	Approximate	Approximate	Approximate	Approximate	Approximate	Approximate	Approximate
MCDB	1302	8.2E-115	2.21E-104	2.64E-111	6.57E-125	3.5E-167	2.46E-146	2.17E-143	1.89E-153		Approximate	Approximate	Approximate	Approximate	Approximate	Approximate	Approximate
	1308	3.7E-114	8.8E-117	1.1E-112	2.29E-129	1.53E-157	3.03E-162	5.61E-145	9.66E-152	3.79E-247		Approximate	Approximate	Approximate	Approximate	Approximate	Approximate
	1309	6.3E-113	6.11E-112	7.68E-118	1.89E-132	5.95E-165	3.65E-158	3.24E-154	2.4E-161	4.18E-257	3.72E-291		Approximate	Approximate	Approximate	Approximate	Approximate
	1314	1.8E-105	4.03E-104	5.49E-109	8.02E-134	6.38E-162	9.41E-150	2.04E-146	3.77E-172	2.96E-239	1.01E-247	1.2E-262		Approximate	Approximate	Approximate	Approximate
MCDB-S	1302	1.65E-95	1.495E-92	3.803E-98	1.78E-113	1.51E-157	8.49E-141	3.94E-136	7.1E-154	5.17E-244	2.78E-226	2.02E-238	1.36E-249		Approximate	Approximate	Approximate
	1308	7.1E-114	3.46E-117	3.09E-114	3.72E-130	3.02E-161	6.25E-167	4.92E-150	5.86E-156	3.86E-237	0	3.34E-281	2.36E-254	1.99E-236		Approximate	Approximate
	1309	8E-114	3.12E-114	8.84E-119	3.99E-133	3.79E-172	9.14E-165	1.65E-162	6.93E-170	5.12E-226	1.67E-247	1.81E-280	6.6E-246	3.6E-263	5.87E-265		Approximate
	1314	1.1E-100	6.6E-101	4.17E-106	6.44E-128	2.25E-164	3.76E-149	9.64E-149	2.7E-175	5.79E-220	1.76E-231	3.05E-241	0	1.86E-266	1.58E-246	1.47E-252	

Supplementary Table S2. Spearman correlation coefficients and p values of secretory miRNA expression signatures of four HUVEC cell lines cultured in four different media. Bold and Italic values are used for calculations in ~~Extended~~ Supplementary Data Figure 1c.

r_s		EGM-2MV				HIMV				MCDB				MCDB-S			
		1302	1308	1309	1314	1302	1308	1309	1314	1302	1308	1309	1314	1302	1308	1309	1314
EGM-2MV	1302	1															
	1308	*0.93925*	1														
	1309	*0.79511*	*0.769291*	1													
	1314	*0.9519*	*0.941967*	*0.8020566*	1												
HIMV	1302	**0.75045**	0.7587755	0.7468811	0.7693909	1											
	1308	0.798415	**0.8343561**	0.7264402	0.8330953	*0.8550291*	1										
	1309	0.682037	0.6818557	**0.7350232**	0.705032	*0.9217471*	*0.7817992*	1									
	1314	0.763977	0.7900967	0.700911	**0.81458**	*0.8897221*	*0.9458226*	*0.8199477*	1								
MCDB	1302	**0.831836**	0.837706	0.8402225	0.848305	**0.7876813**	0.8190696	0.7427478	0.7858951	1							
	1308	0.901964	**0.9123482**	0.7982834	0.9131735	0.7680784	**0.8554395**	0.7139697	0.8118716	*0.8959675*	1						
	1309	0.832886	0.82819	**0.8307166**	0.842335	0.797131	0.8162919	**0.7563404**	0.7915943	*0.9327514*	*0.905786*	1					
	1314	0.857509	0.8914916	0.7931814	**0.9037526**	0.7867351	0.8597824	0.7325297	**0.8394628**	*0.9142651*	*0.9565022*	*0.9081388*	1				
MCDB-S	1302	**0.841767**	0.8717551	0.7556856	0.8644391	**0.799501**	0.8630391	0.7207073	0.8236715	**0.8723903**	0.8897729	0.8755559	0.8886705	1			
	1308	0.847802	**0.8813043**	0.6844963	0.8623838	0.7447979	**0.8195882**	0.6689357	0.7744732	0.7854609	**0.8694214**	0.780252	0.8481488	*0.8794404*	1		
	1309	0.79957	0.7956317	**0.8044021**	0.8370313	0.8065791	0.8112066	**0.7487428**	0.7808372	0.898648	0.8540877	**0.8628826**	0.8683305	*0.894707*	*0.8409495*	1	
	1314	0.843007	0.8795286	0.690769	**0.8797475**	0.7702561	0.8714158	0.688086	**0.8313148**	0.8060023	0.877461	0.796081	**0.8803154**	*0.9014916*	*0.9741716*	*0.8564438*	1

p		EGM-2MV				HIMV				MCDB				MCDB-S			
		1302	1308	1309	1314	1302	1308	1309	1314	1302	1308	1309	1314	1302	1308	1309	1314
EGM-2MV	1302		Approximate	Approximate	Approximate	Approximate	Approximate	Approximate	Approximate	Approximate	Approximate	Approximate	Approximate	Approximate	Approximate	Approximate	Approximate
	1308	5.5E-133		Approximate	Approximate	Approximate	Approximate	Approximate	Approximate	Approximate	Approximate	Approximate	Approximate	Approximate	Approximate	Approximate	Approximate
	1309	3.37E-63	8.338E-57		Approximate	Approximate	Approximate	Approximate	Approximate	Approximate	Approximate	Approximate	Approximate	Approximate	Approximate	Approximate	Approximate
	1314	6.8E-147	1.05E-135	4.436E-65		Approximate	Approximate	Approximate	Approximate	Approximate	Approximate	Approximate	Approximate	Approximate	Approximate	Approximate	Approximate
HIMV	1302	1.21E-52	1.956E-54	6.759E-52	7.906E-57		Approximate	Approximate	Approximate	Approximate	Approximate	Approximate	Approximate	Approximate	Approximate	Approximate	Approximate
	1308	4.38E-64	6.447E-75	7.527E-48	1.707E-74	2.099E-82		Approximate	Approximate	Approximate	Approximate	Approximate	Approximate	Approximate	Approximate	Approximate	Approximate
	1309	3.3E-40	3.525E-40	1.672E-49	5.486E-44	4.97E-118	8.555E-60		Approximate	Approximate	Approximate	Approximate	Approximate	Approximate	Approximate	Approximate	Approximate
	1314	1.36E-55	6.889E-62	2.774E-43	1.154E-68	4.895E-98	8.56E-140	2.763E-70		Approximate	Approximate	Approximate	Approximate	Approximate	Approximate	Approximate	Approximate
MCDB	1302	4.48E-74	4.659E-76	6.22E-77	7.574E-80	2.867E-61	5.132E-70	4.776E-51	8.131E-61		Approximate	Approximate	Approximate	Approximate	Approximate	Approximate	Approximate
	1308	7.5E-105	2.22E-111	4.753E-64	6.21E-112	1.588E-56	1.451E-82	1.479E-45	7.248E-68	2.08E-101		Approximate	Approximate	Approximate	Approximate	Approximate	Approximate
	1309	2E-74	6.989E-73	1.048E-73	1.117E-77	9.709E-64	3.557E-69	6.651E-54	2.819E-62	5.76E-127	3.63E-107		Approximate	Approximate	Approximate	Approximate	Approximate
	1314	2.22E-83	5.7E-99	1.085E-62	6.35E-106	4.986E-61	2.722E-84	5.13E-49	1.147E-76	1.13E-112	6.58E-153	1.22E-108		Approximate	Approximate	Approximate	Approximate
MCDB-S	1302	1.78E-77	2.272E-89	9.221E-54	3.292E-86	2.223E-64	1.263E-85	8.838E-47	1.927E-71	1.182E-89	4.604E-98	4.329E-91	1.727E-97		Approximate	Approximate	Approximate
	1308	1.16E-79	8.413E-94	1.353E-40	2.357E-85	1.819E-51	3.562E-70	3.317E-38	5.083E-58	1.046E-60	2.429E-88	2.054E-59	8.654E-80	6.596E-93		Approximate	Approximate
	1309	2.13E-64	2.442E-63	9.897E-66	7.946E-76	2.414E-66	1.133E-67	2.767E-52	1.476E-59	6.39E-103	4.872E-82	1.466E-85	7.238E-88	1.04E-100	3.454E-77		Approximate
	1314	6.43E-78	5.988E-93	1.334E-41	4.709E-93	4.98E-57	3.215E-89	3.619E-41	6.659E-74	3.515E-66	5.661E-92	1.854E-63	2.519E-93	1.42E-104	2.77E-184	5.854E-83	

Supplementary Table S3. List of HUVEC miRNAs detected in this study, signifying their localisation as intracellular (int-miR) or secretory (sec-miR) or both.

Systematic Name	int-miR	sec-miR	no literature reference in 2023	Systematic Name	int-miR	sec-miR	no literature reference in 2023
hsa-let-7a-5p	1	1		hsa-miR-1224-5p	1	1	
hsa-let-7b-3p		1		hsa-miR-1225-5p	1	1	
hsa-let-7b-5p	1	1		hsa-miR-1226-5p		1	
hsa-let-7c-5p	1	1		hsa-miR-1227-5p	1	1	
hsa-let-7d-3p		1		hsa-miR-1228-3p	1	1	
hsa-let-7d-5p	1	1		hsa-miR-1229-3p		1	
hsa-let-7e-5p	1	1		hsa-miR-1229-5p	1	1	
hsa-let-7f-5p	1	1		hsa-miR-1233-5p	1		
hsa-let-7g-5p	1	1		hsa-miR-1236-5p		1	
hsa-let-7i-5p	1	1		hsa-miR-1246	1	1	
hsa-miR-100-5p	1	1		hsa-miR-1249-3p		1	
hsa-miR-101-3p	1			hsa-miR-1249-5p		1	
hsa-miR-103a-3p	1	1		hsa-miR-1253		1	
hsa-miR-106b-5p	1			hsa-miR-125a-3p	1	1	
hsa-miR-107	1	1		hsa-miR-125a-5p	1	1	
hsa-miR-10a-5p	1	1		hsa-miR-125b-2-3p	1		
hsa-miR-10b-5p	1	1		hsa-miR-125b-5p	1	1	
hsa-miR-1181	1	1		hsa-miR-126-3p	1	1	
hsa-miR-1182		1		hsa-miR-1260b	1	1	
hsa-miR-1183		1		hsa-miR-1268a	1	1	
hsa-miR-1185-1-3p	1	1		hsa-miR-1268b	1	1	
hsa-miR-1185-2-3p	1	1		hsa-miR-127-3p	1		
hsa-miR-1202	1	1		hsa-miR-1273f		1	
hsa-miR-1207-5p	1	1		hsa-miR-1273g-3p	1	1	
hsa-miR-122-5p		1		hsa-miR-1275	1	1	

Systematic Name	int-miR	sec-miR	no literature reference in 2023
hsa-miR-128-3p	1		
hsa-miR-1281		1	
hsa-miR-1288-3p	1		
hsa-miR-1290	1	1	
hsa-miR-1295a		1	
hsa-miR-1299		1	
hsa-miR-1305	1	1	
hsa-miR-130a-3p	1	1	
hsa-miR-130b-3p	1	1	
hsa-miR-132-3p	1		
hsa-miR-1322		1	
hsa-miR-133b		1	
hsa-miR-134-5p	1	1	
hsa-miR-1343-5p		1	
hsa-miR-135a-3p	1	1	
hsa-miR-136-3p	1		
hsa-miR-136-5p	1		
hsa-miR-138-5p		1	
hsa-miR-139-3p		1	
hsa-miR-140-3p	1		
hsa-miR-140-5p	1		
hsa-miR-146a-5p	1	1	
hsa-miR-146b-5p	1		
hsa-miR-1470		1	
hsa-miR-1471		1	
hsa-miR-148a-3p	1		
hsa-miR-148b-3p	1		
hsa-miR-150-3p	1	1	

Systematic Name	int-miR	sec-miR	no literature reference in 2023
hsa-miR-151a-3p	1	1	
hsa-miR-151a-5p	1	1	
hsa-miR-151b	1	1	
hsa-miR-152-3p	1		
hsa-miR-154-3p	1		
hsa-miR-154-5p	1		
hsa-miR-155-5p	1		
hsa-miR-1587	1	1	
hsa-miR-15a-5p	1	1	
hsa-miR-15b-5p	1	1	
hsa-miR-16-5p	1	1	
hsa-miR-17-3p	1		
hsa-miR-17-5p	1	1	
hsa-miR-181a-2-3p	1		
hsa-miR-181a-3p	1		
hsa-miR-181a-5p	1	1	
hsa-miR-181b-5p	1	1	
hsa-miR-181c-5p	1		
hsa-miR-181d-5p	1		
hsa-miR-1825		1	
hsa-miR-185-5p	1		
hsa-miR-186-5p	1		
hsa-miR-188-5p	1	1	
hsa-miR-18a-5p	1		
hsa-miR-18b-5p	1		
hsa-miR-1908-3p	1	1	
hsa-miR-191-3p		1	
hsa-miR-1914-3p	1	1	

Systematic Name	int-miR	sec-miR	no literature reference in 2023	Systematic Name	int-miR	sec-miR	no literature reference in 2023
hsa-miR-1915-3p	1	1		hsa-miR-218-5p	1		
hsa-miR-192-5p	1			hsa-miR-22-3p	1	1	
hsa-miR-193a-3p	1			hsa-miR-22-5p	1		
hsa-miR-193a-5p	1	1		hsa-miR-221-3p	1	1	
hsa-miR-193b-3p	1			hsa-miR-221-5p	1		
hsa-miR-193b-5p		1		hsa-miR-222-3p	1		
hsa-miR-195-5p	1			hsa-miR-224-5p	1		
hsa-miR-196b-5p	1			hsa-miR-2276-3p		1	
hsa-miR-197-5p	1	1		hsa-miR-2392	1	1	
hsa-miR-1973	1			hsa-miR-23a-3p	1	1	
hsa-miR-199a-3p	1	1		hsa-miR-23b-3p	1	1	
hsa-miR-199a-5p	1			hsa-miR-24-1-5p	1		
hsa-miR-19a-3p	1			hsa-miR-24-3p	1	1	
hsa-miR-19b-3p	1	1		hsa-miR-25-3p	1	1	
hsa-miR-202-3p		1		hsa-miR-26a-5p	1		
hsa-miR-204-5p	1			hsa-miR-26b-5p	1		
hsa-miR-208a-5p	1	1		hsa-miR-27a-3p	1	1	
hsa-miR-20a-5p	1	1		hsa-miR-27b-3p	1	1	
hsa-miR-20b-5p	1			hsa-miR-28-5p	1		
hsa-miR-21-3p	1			hsa-miR-2861	1	1	
hsa-miR-21-5p	1	1		hsa-miR-299-3p	1		
hsa-miR-210-3p	1			hsa-miR-299-5p	1		
hsa-miR-211-3p	1	1		hsa-miR-29a-3p	1	1	
hsa-miR-2117		1		hsa-miR-29b-1-5p	1		
hsa-miR-212-3p	1			hsa-miR-29b-3p	1		
hsa-miR-214-3p	1	1		hsa-miR-29c-3p	1		
hsa-miR-216a-5p	1			hsa-miR-301a-3p	1		
hsa-miR-217	1			hsa-miR-301b-3p	1		

Systematic Name	int-miR	sec-miR	no literature reference in 2023	Systematic Name	int-miR	sec-miR	no literature reference in 2023
hsa-miR-30a-3p	1			hsa-miR-320a	1	1	
hsa-miR-30a-5p	1	1		hsa-miR-320b	1	1	
hsa-miR-30b-5p	1			hsa-miR-320c	1	1	
hsa-miR-30c-5p	1			hsa-miR-320d	1	1	
hsa-miR-30d-5p	1	1		hsa-miR-320e	1	1	
hsa-miR-30e-3p	1			hsa-miR-323a-3p	1		
hsa-miR-30e-5p	1			hsa-miR-324-3p	1	1	
hsa-miR-31-3p	1			hsa-miR-324-5p	1		
hsa-miR-31-5p	1			hsa-miR-328-3p		1	
hsa-miR-3125	1	1		hsa-miR-328-5p	1	1	
hsa-miR-3127-5p	1	1		hsa-miR-331-3p	1	1	
hsa-miR-3129-3p		1		hsa-miR-335-5p	1		
hsa-miR-3132	1			hsa-miR-337-5p	1		
hsa-miR-3135b	1	1		hsa-miR-338-3p	1		
hsa-miR-3138		1		hsa-miR-339-3p		1	
hsa-miR-3141	1	1		hsa-miR-33a-5p	1		
hsa-miR-3152-3p		1		hsa-miR-340-5p	1		
hsa-miR-3156-5p	1	1		hsa-miR-342-3p	1	1	
hsa-miR-3162-3p		1		hsa-miR-345-3p		1	
hsa-miR-3162-5p	1	1		hsa-miR-34a-5p	1	1	
hsa-miR-3184-3p		1		hsa-miR-34b-5p	1		
hsa-miR-3186-3p		1		hsa-miR-361-3p	1		
hsa-miR-3188	1	1		hsa-miR-361-5p	1	1	
hsa-miR-3194-5p		1		hsa-miR-3610		1	
hsa-miR-3195	1	1		hsa-miR-362-3p	1		
hsa-miR-3196	1	1		hsa-miR-362-5p	1		
hsa-miR-3198	1	1		hsa-miR-3620-3p		1	
hsa-miR-3202		1		hsa-miR-3621		1	

Systematic Name	int-miR	sec-miR	no literature reference in 2023	Systematic Name	int-miR	sec-miR	no literature reference in 2023
hsa-miR-3648	1	1		hsa-miR-378g		1	
hsa-miR-3651	1			hsa-miR-378i	1		
hsa-miR-3652	1	1		hsa-miR-379-5p	1		
hsa-miR-3653-3p	1			hsa-miR-381-3p	1		
hsa-miR-3656	1	1		hsa-miR-382-5p	1		
hsa-miR-3659	1			hsa-miR-3911	1		
hsa-miR-365a-3p	1	1		hsa-miR-3937	1	1	
hsa-miR-3663-3p	1	1		hsa-miR-3938		1	
hsa-miR-3665	1	1		hsa-miR-3940-5p	1	1	
hsa-miR-3667-5p		1		hsa-miR-3960	1	1	
hsa-miR-3675-3p		1		hsa-miR-409-3p	1		
hsa-miR-3679-5p	1	1		hsa-miR-409-5p	1		
hsa-miR-3682-3p		1		hsa-miR-410-3p	1		
hsa-miR-3689f		1		hsa-miR-411-5p	1		
hsa-miR-369-5p	1			hsa-miR-421	1		
hsa-miR-3692-5p		1		hsa-miR-422a		1	
hsa-miR-370-3p	1			hsa-miR-423-5p	1	1	
hsa-miR-371a-5p	1	1		hsa-miR-424-5p	1	1	
hsa-miR-371b-5p	1	1		hsa-miR-425-3p		1	
hsa-miR-373-5p		1		hsa-miR-425-5p	1		
hsa-miR-374a-5p	1			hsa-miR-4257	1	1	
hsa-miR-374b-5p	1			hsa-miR-4270	1	1	
hsa-miR-376a-3p	1			hsa-miR-4271	1	1	
hsa-miR-376a-5p	1			hsa-miR-4274		1	
hsa-miR-376b-3p	1			hsa-miR-4281	1	1	
hsa-miR-376c-3p	1			hsa-miR-4284	1		
hsa-miR-377-3p	1			hsa-miR-4286	1	1	
hsa-miR-378f		1		hsa-miR-4291	1		

Systematic Name	int-miR	sec-miR	no literature reference in 2023	Systematic Name	int-miR	sec-miR	no literature reference in 2023
hsa-miR-4298		1		hsa-miR-4485-3p	1	1	
hsa-miR-4299	1	1		hsa-miR-4485-5p	1	1	
hsa-miR-4306	1	1		hsa-miR-4488		1	
hsa-miR-431-5p	1			hsa-miR-4496		1	
hsa-miR-4317	1			hsa-miR-4497	1	1	
hsa-miR-432-5p	1			hsa-miR-4499	1	1	
hsa-miR-4322		1		hsa-miR-4505	1	1	
hsa-miR-4324	1			hsa-miR-4507	1	1	
hsa-miR-4327	1	1		hsa-miR-4508		1	
hsa-miR-4417		1		hsa-miR-4514		1	
hsa-miR-4419b		1		hsa-miR-4515	1	1	1
hsa-miR-4428	1			hsa-miR-4516	1	1	
hsa-miR-4429		1		hsa-miR-451a		1	
hsa-miR-4430	1	1		hsa-miR-4530	1	1	
hsa-miR-4433a-3p		1		hsa-miR-4532	1	1	
hsa-miR-4433b-3p		1		hsa-miR-4534		1	
hsa-miR-4442	1	1		hsa-miR-454-3p	1		
hsa-miR-4443	1	1		hsa-miR-455-3p	1		
hsa-miR-4449	1			hsa-miR-4632-5p		1	
hsa-miR-4459	1	1		hsa-miR-4634	1	1	
hsa-miR-4462	1		1	hsa-miR-4644		1	
hsa-miR-4463	1	1		hsa-miR-4646-5p		1	
hsa-miR-4465	1	1		hsa-miR-4649-3p		1	
hsa-miR-4466	1	1		hsa-miR-4651		1	
hsa-miR-4472		1		hsa-miR-4653-3p	1	1	
hsa-miR-4476		1		hsa-miR-4655-3p	1	1	
hsa-miR-4478	1	1		hsa-miR-4656	1		
hsa-miR-4484	1	1		hsa-miR-4659a-3p		1	

Systematic Name	int-miR	sec-miR	no literature reference in 2023	Systematic Name	int-miR	sec-miR	no literature reference in 2023
hsa-miR-4668-5p	1			hsa-miR-4782-3p		1	
hsa-miR-4669	1	1		hsa-miR-4785		1	
hsa-miR-4672	1	1		hsa-miR-4787-3p	1	1	1
hsa-miR-4673		1		hsa-miR-4787-5p	1	1	
hsa-miR-4687-3p	1	1		hsa-miR-4788	1	1	1
hsa-miR-4689	1	1		hsa-miR-4800-5p		1	1
hsa-miR-4698		1		hsa-miR-483-3p		1	
hsa-miR-4701-5p		1		hsa-miR-483-5p	1	1	
hsa-miR-4707-5p		1		hsa-miR-484	1		
hsa-miR-4713-3p	1	1		hsa-miR-487a-3p	1		
hsa-miR-4716-3p	1	1	1	hsa-miR-487b-3p	1		
hsa-miR-4716-5p		1		hsa-miR-493-5p	1		
hsa-miR-4719		1		hsa-miR-494-3p	1	1	
hsa-miR-4721	1	1		hsa-miR-495-3p	1		
hsa-miR-4725-3p		1		hsa-miR-497-5p	1		
hsa-miR-4725-5p		1		hsa-miR-498		1	
hsa-miR-4728-5p	1	1		hsa-miR-5001-5p	1	1	
hsa-miR-4734		1		hsa-miR-5006-5p	1	1	
hsa-miR-4738-3p		1		hsa-miR-500a-3p	1		
hsa-miR-4739	1	1		hsa-miR-500a-5p	1		
hsa-miR-4741	1	1		hsa-miR-503-5p	1		
hsa-miR-4745-5p	1	1		hsa-miR-504-3p		1	
hsa-miR-4746-3p	1	1	1	hsa-miR-505-3p	1		
hsa-miR-4762-5p		1		hsa-miR-5088-5p	1	1	
hsa-miR-4763-3p	1	1		hsa-miR-5100	1	1	
hsa-miR-4767		1		hsa-miR-513a-3p		1	
hsa-miR-4769-3p		1		hsa-miR-513a-5p	1	1	
hsa-miR-4778-5p		1		hsa-miR-515-5p		1	

Systematic Name	int-miR	sec-miR	no literature reference in 2023	Systematic Name	int-miR	sec-miR	no literature reference in 2023
hsa-miR-5194	1	1		hsa-miR-5787	1	1	
hsa-miR-5195-3p	1	1		hsa-miR-590-5p	1		
hsa-miR-5196-5p		1		hsa-miR-595		1	
hsa-miR-519b-3p		1		hsa-miR-601		1	
hsa-miR-532-3p	1			hsa-miR-602		1	
hsa-miR-532-5p	1			hsa-miR-6068	1	1	
hsa-miR-539-5p	1			hsa-miR-6069		1	
hsa-miR-542-3p	1			hsa-miR-6075		1	
hsa-miR-543	1			hsa-miR-6076	1	1	
hsa-miR-548ae-3p	1		1	hsa-miR-6085	1	1	
hsa-miR-548ap-3p	1			hsa-miR-6086		1	
hsa-miR-548c-3p	1			hsa-miR-6087	1	1	
hsa-miR-548f-3p	1		1	hsa-miR-6088	1	1	
hsa-miR-548q		1		hsa-miR-6089	1	1	
hsa-miR-548x-3p	1			hsa-miR-6090	1	1	
hsa-miR-557		1		hsa-miR-6124	1	1	
hsa-miR-5581-5p	1	1	1	hsa-miR-6125	1	1	
hsa-miR-5585-3p		1		hsa-miR-6126	1	1	
hsa-miR-5586-3p		1		hsa-miR-6127	1	1	
hsa-miR-5587-5p		1		hsa-miR-6131	1	1	
hsa-miR-5684	1			hsa-miR-6132	1		
hsa-miR-5685		1	1	hsa-miR-6165	1	1	
hsa-miR-5703		1		hsa-miR-625-5p	1		
hsa-miR-572	1	1		hsa-miR-629-3p	1		
hsa-miR-5739	1	1		hsa-miR-630		1	
hsa-miR-574-3p	1	1		hsa-miR-636		1	
hsa-miR-574-5p	1	1		hsa-miR-638	1	1	
hsa-miR-575	1	1		hsa-miR-642a-3p	1	1	

Systematic Name	int-miR	sec-miR	no literature reference in 2023	Systematic Name	int-miR	sec-miR	no literature reference in 2023
hsa-miR-642b-3p	1	1		hsa-miR-6752-5p	1	1	
hsa-miR-643	1			hsa-miR-6755-3p		1	
hsa-miR-6500-5p	1	1		hsa-miR-6756-5p	1	1	
hsa-miR-6508-5p	1	1		hsa-miR-6757-5p		1	1
hsa-miR-6510-5p		1	1	hsa-miR-6760-5p		1	
hsa-miR-6512-5p	1	1	1	hsa-miR-6763-5p	1	1	
hsa-miR-6515-3p	1	1		hsa-miR-6765-3p		1	
hsa-miR-6516-3p	1			hsa-miR-6767-5p	1	1	1
hsa-miR-652-3p	1			hsa-miR-6768-5p	1	1	
hsa-miR-654-3p	1			hsa-miR-6769b-5p	1	1	
hsa-miR-660-5p	1			hsa-miR-6775-5p		1	1
hsa-miR-663a		1		hsa-miR-6776-5p		1	
hsa-miR-664a-3p	1			hsa-miR-6777-3p		1	
hsa-miR-664b-3p	1			hsa-miR-6777-5p		1	
hsa-miR-671-5p		1		hsa-miR-6779-5p		1	
hsa-miR-6717-5p	1	1		hsa-miR-6780a-5p	1	1	
hsa-miR-6722-3p		1		hsa-miR-6780b-5p	1	1	
hsa-miR-6723-5p	1	1		hsa-miR-6784-5p		1	
hsa-miR-6724-5p	1	1		hsa-miR-6785-5p	1	1	
hsa-miR-6727-5p	1	1		hsa-miR-6786-5p		1	1
hsa-miR-6728-5p	1	1	1	hsa-miR-6789-5p	1	1	1
hsa-miR-6734-5p	1	1		hsa-miR-6790-5p		1	1
hsa-miR-6737-5p		1		hsa-miR-6791-5p	1	1	
hsa-miR-6738-5p		1		hsa-miR-6792-5p		1	
hsa-miR-6739-5p		1		hsa-miR-6793-5p		1	
hsa-miR-6740-5p	1	1		hsa-miR-6794-5p		1	
hsa-miR-6741-5p		1		hsa-miR-6800-5p	1	1	1
hsa-miR-6749-5p	1	1	1	hsa-miR-6802-5p	1	1	1

Systematic Name	int-miR	sec-miR	no literature reference in 2023	Systematic Name	int-miR	sec-miR	no literature reference in 2023
hsa-miR-6803-5p	1	1		hsa-miR-7-5p	1		
hsa-miR-6807-5p	1	1		hsa-miR-7106-5p		1	
hsa-miR-6808-5p	1	1		hsa-miR-7107-5p	1	1	
hsa-miR-6812-5p	1	1	1	hsa-miR-7108-5p	1	1	
hsa-miR-6819-5p		1		hsa-miR-711		1	
hsa-miR-6820-5p	1			hsa-miR-7110-5p	1	1	
hsa-miR-6821-5p	1	1		hsa-miR-7111-3p		1	
hsa-miR-6824-5p		1		hsa-miR-7114-5p	1	1	
hsa-miR-6826-5p	1	1		hsa-miR-7150	1	1	
hsa-miR-6829-5p	1	1	1	hsa-miR-7152-3p	1	1	1
hsa-miR-6831-5p		1		hsa-miR-7158-3p		1	
hsa-miR-6833-5p		1	1	hsa-miR-718		1	
hsa-miR-6840-3p	1	1		hsa-miR-744-5p	1		
hsa-miR-6848-3p		1		hsa-miR-758-3p	1		
hsa-miR-6849-5p		1	1	hsa-miR-760		1	
hsa-miR-6850-5p	1	1		hsa-miR-762	1	1	
hsa-miR-6858-5p		1		hsa-miR-7641	1	1	
hsa-miR-6865-3p		1		hsa-miR-765		1	
hsa-miR-6867-5p		1		hsa-miR-769-5p	1		
hsa-miR-6869-5p	1	1		hsa-miR-770-5p	1		
hsa-miR-6870-5p		1		hsa-miR-7704	1	1	
hsa-miR-6872-3p	1	1		hsa-miR-7845-5p		1	1
hsa-miR-6875-5p	1	1		hsa-miR-7846-3p		1	1
hsa-miR-6879-5p	1	1		hsa-miR-7847-3p	1	1	
hsa-miR-6887-5p	1			hsa-miR-7851-3p		1	
hsa-miR-6891-3p		1		hsa-miR-7975	1	1	
hsa-miR-6891-5p	1	1		hsa-miR-7977	1	1	
hsa-miR-6893-5p	1	1	1	hsa-miR-8063	1	1	

Systematic Name	int-miR	sec-miR	no literature reference in 2023
hsa-miR-8064		1	
hsa-miR-8069	1	1	1
hsa-miR-8072	1	1	
hsa-miR-8085		1	
hsa-miR-8087		1	
hsa-miR-8089		1	
hsa-miR-8485		1	
hsa-miR-874-3p	1	1	
hsa-miR-875-3p		1	
hsa-miR-876-3p		1	
hsa-miR-885-5p		1	
hsa-miR-887-3p	1	1	
hsa-miR-892b	1		
hsa-miR-92a-3p	1	1	
hsa-miR-93-5p	1	1	
hsa-miR-937-5p	1	1	
hsa-miR-939-5p	1	1	
hsa-miR-98-5p	1		
hsa-miR-99a-5p	1	1	
hsa-miR-99b-5p	1	1	

Supplementary Table S4. List of HUVEC miRNAs detected in time-course experiment. Localisation is shown as intracellular (int-miR), secretory (sec-miR) or both along with their correlation with duration in culture.

SystematicName	int-miR	sec-miR	int-miR Pearson r	int-miR p-value	sec-miR Pearson r	sec-miR p-value
hsa-let-7a-5p	1	1	0.5054	0.385	-0.07596	0.9034
hsa-let-7b-5p	1	1	0.339	0.5768	0.6467	0.2382
hsa-let-7c-5p	1	1	0.5426	0.3447	0.165	0.7909
hsa-let-7d-5p	1		0.3701	0.5397		
hsa-let-7e-5p	1		0.2972	0.6273		
hsa-let-7f-5p	1	1	0.623	0.2616	0.507	0.3833
hsa-let-7g-5p	1		0.4708	0.4235		
hsa-let-7i-5p	1		0.4291	0.4709		
hsa-miR-100-5p	1		0.4638	0.4314		
hsa-miR-101-3p	1		-0.6747	0.2115		
hsa-miR-103a-3p	1		-0.04193	0.9466		
hsa-miR-106b-5p	1		-0.01663	0.9788		
hsa-miR-107	1		0.2161	0.7271		
hsa-miR-10a-5p	1		-0.4273	0.473		
hsa-miR-10b-5p	1		-0.192	0.757		
hsa-miR-1181	1		0.8411	0.0742		
hsa-miR-1185-1-3p	1		0.5002	0.3908		
hsa-miR-1185-2-3p	1		0.8055	0.0999		
hsa-miR-1202	1	1	0.8871	0.0448	-0.2329	0.7061
hsa-miR-1207-5p	1	1	0.9918	0.0009	0.7721	0.1261
hsa-miR-1224-5p	1		0.9898	0.0012		
hsa-miR-1225-5p	1	1	0.9956	0.0004	-0.545	0.3421
hsa-miR-1227-5p	1	1	0.9874	0.0017	0.6983	0.1896
hsa-miR-1229-5p	1	1	0.9685	0.0067	0.7819	0.1182
hsa-miR-1233-5p	1		0.5819	0.3034		
hsa-miR-1236-5p		1			-0.5675	0.3184

SystematicName	int-miR	sec-miR	int-miR Pearson r	int-miR p-value	sec-miR Pearson r	sec-miR p-value
hsa-miR-1246	1		-0.1128	0.8567		
hsa-miR-125a-3p	1		0.8731	0.0532		
hsa-miR-125a-5p	1		-0.4091	0.4941		
hsa-miR-125b-2-3p	1		0.1911	0.7582		
hsa-miR-125b-5p	1	1	-0.3543	0.5585	-0.02347	0.9701
hsa-miR-126-3p	1	1	-0.6296	0.255	0.7369	0.1555
hsa-miR-1260b	1		-0.1962	0.7518		
hsa-miR-1268a	1	1	0.8726	0.0535	-0.7283	0.1629
hsa-miR-1268b	1	1	0.9529	0.0122	0.6764	0.2099
hsa-miR-127-3p	1		-0.4767	0.4169		
hsa-miR-1273g-3p	1	1	-0.2368	0.7014	-0.6713	0.2147
hsa-miR-1275	1		0.967	0.0072		
hsa-miR-128-3p	1		-0.6382	0.2465		
hsa-miR-1281	1		-0.2511	0.6836		
hsa-miR-1288-3p	1		0.003923	0.995		
hsa-miR-1299		1			-0.03209	0.9591
hsa-miR-1305	1		-0.4412	0.457		
hsa-miR-130a-3p	1		-0.08467	0.8923		
hsa-miR-130b-3p	1		0.08437	0.8927		
hsa-miR-134-5p	1		0.9961	0.0003		
hsa-miR-135a-3p	1		0.8982	0.0384		
hsa-miR-136-3p	1		0.636	0.2487		
hsa-miR-136-5p	1		-0.4938	0.3978		
hsa-miR-137	1		-0.03993	0.9492		
hsa-miR-140-3p	1		-0.8832	0.0471		
hsa-miR-140-5p	1		-0.4308	0.4689		
hsa-miR-146a-5p	1		-0.5961	0.2887		
hsa-miR-148a-3p	1		-0.6385	0.2463		
hsa-miR-148b-3p	1		-0.5071	0.3832		
hsa-miR-150-3p	1		0.8982	0.0384		

SystematicName	int-miR	sec-miR	int-miR Pearson r	int-miR p-value	sec-miR Pearson r	sec-miR p-value
hsa-miR-151a-3p	1		-0.5095	0.3806		
hsa-miR-151a-5p	1		0.5433	0.344		
hsa-miR-151b	1		0.2591	0.6739		
hsa-miR-152-3p	1		-0.02842	0.9638		
hsa-miR-154-3p	1		-0.8125	0.0947		
hsa-miR-154-5p	1		-0.3809	0.527		
hsa-miR-155-5p	1		-0.392	0.514		
hsa-miR-1587	1	1	0.5749	0.3107	0.7156	0.1741
hsa-miR-15a-5p	1		-0.3542	0.5587		
hsa-miR-15b-5p	1		0.4812	0.4118		
hsa-miR-16-5p	1		0.3271	0.5911		
hsa-miR-17-3p	1		-0.4729	0.4211		
hsa-miR-17-5p	1		-0.09851	0.8748		
hsa-miR-181a-2-3p	1		0.1181	0.85		
hsa-miR-181a-3p	1		0.2656	0.6659		
hsa-miR-181a-5p	1		-0.7884	0.1131		
hsa-miR-181b-5p	1		-0.5597	0.3265		
hsa-miR-181c-5p	1		-0.4766	0.417		
hsa-miR-181d-5p	1		-0.4617	0.4338		
hsa-miR-185-5p	1		0.14	0.8224		
hsa-miR-188-5p	1		0.8982	0.0384		
hsa-miR-18a-5p	1		-0.3016	0.6219		
hsa-miR-18b-5p	1		-0.2559	0.6777		
hsa-miR-1914-3p	1		0.4468	0.4507		
hsa-miR-1915-3p	1	1	0.9127	0.0305	-0.9187	0.0275
hsa-miR-192-5p	1		0.8982	0.0384		
hsa-miR-193a-3p	1		-0.3724	0.537		
hsa-miR-193a-5p	1		0.712	0.1773		
hsa-miR-193b-3p	1		-0.6378	0.2469		
hsa-miR-195-5p	1		-0.01143	0.9854		

SystematicName	int-miR	sec-miR	int-miR Pearson r	int-miR p-value	sec-miR Pearson r	sec-miR p-value
hsa-miR-196b-5p	1		-0.4502	0.4468		
hsa-miR-197-5p	1		-0.7574	0.1381		
hsa-miR-1973	1		0.7248	0.166		
hsa-miR-199a-3p	1		0.1733	0.7805		
hsa-miR-199a-5p	1		-0.4807	0.4125		
hsa-miR-19a-3p	1		-0.3713	0.5383		
hsa-miR-19b-3p	1		-0.2983	0.6259		
hsa-miR-204-5p	1		-0.6993	0.1888		
hsa-miR-208a-5p	1		-0.4701	0.4242		
hsa-miR-20a-5p	1		0.008193	0.9896		
hsa-miR-20b-5p	1		-0.4708	0.4235		
hsa-miR-21-3p	1		0.7255	0.1654		
hsa-miR-21-5p	1	1	-0.6845	0.2024	-0.8321	0.0805
hsa-miR-210-3p	1		-0.4892	0.403		
hsa-miR-211-3p	1		-0.1192	0.8486		
hsa-miR-214-3p	1		-0.6469	0.2381		
hsa-miR-216a-5p	1		-0.6581	0.2273		
hsa-miR-218-5p	1		-0.5944	0.2905		
hsa-miR-22-3p	1		-0.8987	0.0381		
hsa-miR-22-5p	1		-0.6872	0.1999		
hsa-miR-221-3p	1		-0.4168	0.4851		
hsa-miR-221-5p	1		0.4742	0.4197		
hsa-miR-222-3p	1		-0.5313	0.3568		
hsa-miR-224-5p	1		0.343	0.5721		
hsa-miR-2392	1		-0.1604	0.7966		
hsa-miR-23a-3p	1	1	-0.8496	0.0684	0.06383	0.9188
hsa-miR-23b-3p	1		-0.4388	0.4598		
hsa-miR-24-1-5p	1		0.6206	0.264		
hsa-miR-24-3p	1	1	-0.4055	0.4982	0.66	0.2255
hsa-miR-25-3p	1		-0.3689	0.5412		

SystematicName	int-miR	sec-miR	int-miR Pearson r	int-miR p-value	sec-miR Pearson r	sec-miR p-value
hsa-miR-26a-5p	1		-0.6747	0.2115		
hsa-miR-26b-5p	1		-0.3149	0.6058		
hsa-miR-27a-3p	1		0.436	0.463		
hsa-miR-27b-3p	1		-0.1504	0.8092		
hsa-miR-28-5p	1		0.1016	0.8708		
hsa-miR-2861	1	1	0.9817	0.003	-0.9096	0.0322
hsa-miR-299-3p	1		-0.8917	0.0421		
hsa-miR-299-5p	1		0.109	0.8615		
hsa-miR-29a-3p	1		0.6129	0.2717		
hsa-miR-29b-1-5p	1		0.9086	0.0327		
hsa-miR-29b-3p	1		-0.5063	0.3841		
hsa-miR-29c-3p	1		-0.7509	0.1436		
hsa-miR-301a-3p	1		-0.2055	0.7403		
hsa-miR-301b-3p	1		0.07596	0.9034		
hsa-miR-30a-3p	1		-0.115	0.8539		
hsa-miR-30a-5p	1		-0.4706	0.4238		
hsa-miR-30b-5p	1		-0.332	0.5852		
hsa-miR-30c-5p	1		-0.3941	0.5116		
hsa-miR-30d-5p	1		-0.5094	0.3807		
hsa-miR-30e-3p	1		0.1398	0.8225		
hsa-miR-30e-5p	1		-0.3919	0.5141		
hsa-miR-31-3p	1		0.9025	0.036		
hsa-miR-31-5p	1		0.1174	0.8509		
hsa-miR-3125	1		-0.2244	0.7167		
hsa-miR-3132	1		0.6834	0.2034		
hsa-miR-3135b	1	1	0.4719	0.4223	0.4645	0.4306
hsa-miR-3151-3p	1		0.6466	0.2383		
hsa-miR-3162-3p	1		-0.2737	0.6559		
hsa-miR-3162-5p	1		0.9754	0.0046		
hsa-miR-3188	1		0.4239	0.477		

SystematicName	int-miR	sec-miR	int-miR Pearson r	int-miR p-value	sec-miR Pearson r	sec-miR p-value
hsa-miR-3195	1		0.8308	0.0814		
hsa-miR-3196	1	1	0.9609	0.0092	0.6849	0.202
hsa-miR-3198	1		-0.6197	0.2649		
hsa-miR-320a	1		0.3416	0.5737		
hsa-miR-320b	1		0.09456	0.8798		
hsa-miR-320c	1		-0.05911	0.9248		
hsa-miR-320d	1		0.02138	0.9728		
hsa-miR-320e	1		0.00875	0.9889		
hsa-miR-323a-3p	1		0.2556	0.6781		
hsa-miR-324-3p	1		-0.8384	0.0761		
hsa-miR-324-5p	1		-0.7309	0.1606		
hsa-miR-329-3p	1		-0.4766	0.417		
hsa-miR-331-3p	1		-0.128	0.8375		
hsa-miR-335-5p	1		-0.6994	0.1887		
hsa-miR-337-5p	1		-0.6432	0.2416		
hsa-miR-338-3p	1		0.09044	0.885		
hsa-miR-340-5p	1		0.1181	0.85		
hsa-miR-342-3p	1		-0.4286	0.4715		
hsa-miR-34a-5p	1		-0.2464	0.6895		
hsa-miR-34b-5p	1		-0.2058	0.7398		
hsa-miR-361-3p	1		0.0008012	0.999		
hsa-miR-361-5p	1		-0.466	0.4289		
hsa-miR-362-3p	1		-0.2016	0.745		
hsa-miR-362-5p	1		0.1987	0.7486		
hsa-miR-3651	1		0.6006	0.2842		
hsa-miR-3652		1			0.8982	0.0384
hsa-miR-3653-3p	1		0.3222	0.597		
hsa-miR-3656	1		0.9171	0.0283		
hsa-miR-3659	1		0.3718	0.5377		
hsa-miR-365a-3p	1		-0.4987	0.3925		

SystematicName	int-miR	sec-miR	int-miR Pearson r	int-miR p-value	sec-miR Pearson r	sec-miR p-value
hsa-miR-3663-3p	1		0.1676	0.7876		
hsa-miR-3665	1	1	0.9872	0.0017	-0.8276	0.0837
hsa-miR-3679-5p	1	1	0.4535	0.4431	0.8982	0.0384
hsa-miR-369-5p	1		-0.5873	0.2978		
hsa-miR-370-3p	1		-0.3792	0.529		
hsa-miR-371a-5p	1		0.8443	0.072		
hsa-miR-371b-5p		1			0.597	0.2878
hsa-miR-374a-5p	1		-0.4764	0.4172		
hsa-miR-374b-5p	1		-0.5217	0.3673		
hsa-miR-376a-3p	1		-0.7656	0.1313		
hsa-miR-376a-5p	1		-0.4509	0.446		
hsa-miR-376b-3p	1		-0.6279	0.2567		
hsa-miR-376c-3p	1		0.02783	0.9646		
hsa-miR-377-3p	1		-0.4459	0.4516		
hsa-miR-378i	1		-0.0259	0.967		
hsa-miR-379-5p	1		-0.6485	0.2366		
hsa-miR-381-3p	1		-0.06127	0.922		
hsa-miR-382-5p	1		-0.3538	0.5591		
hsa-miR-3911	1		0.636	0.2487		
hsa-miR-3926	1		-0.2016	0.745		
hsa-miR-3940-5p	1	1	0.9841	0.0024	0.8982	0.0384
hsa-miR-3960	1	1	0.9897	0.0013	0.9246	0.0246
hsa-miR-409-3p	1		-0.1361	0.8272		
hsa-miR-409-5p	1		-0.462	0.4335		
hsa-miR-410-3p	1		-0.3682	0.542		
hsa-miR-411-5p	1		0.07123	0.9094		
hsa-miR-423-5p	1		-0.2254	0.7154		
hsa-miR-424-5p	1		-0.2998	0.6241		
hsa-miR-425-5p	1		-0.6706	0.2153		
hsa-miR-4257	1		0.9234	0.0252		

SystematicName	int-miR	sec-miR	int-miR Pearson r	int-miR p-value	sec-miR Pearson r	sec-miR p-value
hsa-miR-4271	1		0.8319	0.0806		
hsa-miR-4281	1	1	0.9623	0.0087	-0.3653	0.5455
hsa-miR-4284	1		0.2673	0.6638		
hsa-miR-4286	1	1	-0.7855	0.1153	-0.8208	0.0886
hsa-miR-4291	1		0.03752	0.9522		
hsa-miR-4298		1			0.165	0.7909
hsa-miR-4299	1	1	-0.4532	0.4434	0.8254	0.0853
hsa-miR-4306	1		0.04244	0.946		
hsa-miR-431-5p	1		-0.1292	0.8359		
hsa-miR-4317	1		-0.6249	0.2597		
hsa-miR-432-5p	1		-0.2694	0.6612		
hsa-miR-4324	1		0.008873	0.9887		
hsa-miR-4327	1		0.8982	0.0384		
hsa-miR-4428	1		0.3015	0.622		
hsa-miR-4442	1		0.08139	0.8965		
hsa-miR-4443	1		0.457	0.4391		
hsa-miR-4449	1		-0.3285	0.5894		
hsa-miR-4459	1	1	0.9543	0.0116	0.9575	0.0105
hsa-miR-4463	1		0.988	0.0016		
hsa-miR-4465	1	1	0.6459	0.2391	0.8982	0.0384
hsa-miR-4466	1	1	0.9847	0.0023	0.943	0.0162
hsa-miR-4472	1		0.3476	0.5666		
hsa-miR-4481	1		-0.4766	0.417		
hsa-miR-4484	1		0.4708	0.4235		
hsa-miR-4485-3p	1		0.9483	0.014		
hsa-miR-4485-5p	1		0.5614	0.3248		
hsa-miR-4497	1		0.984	0.0024		
hsa-miR-4499	1	1	0.9743	0.0049	0.8982	0.0384
hsa-miR-4505	1	1	0.9306	0.0217	0.627	0.2576
hsa-miR-4507	1	1	0.8885	0.0439	0.8485	0.0691

SystematicName	int-miR	sec-miR	int-miR Pearson r	int-miR p-value	sec-miR Pearson r	sec-miR p-value
hsa-miR-4515	1		0.001239	0.9984		
hsa-miR-4516	1	1	-0.8577	0.0631	0.8215	0.0881
hsa-miR-4530	1	1	0.957	0.0106	0.9478	0.0142
hsa-miR-4532	1		0.8941	0.0407		
hsa-miR-454-3p	1		-0.4132	0.4893		
hsa-miR-455-3p	1		-0.04052	0.9484		
hsa-miR-4634	1		0.6912	0.1962		
hsa-miR-4649-3p	1		-0.3041	0.6188		
hsa-miR-4652-3p	1		0.5278	0.3606		
hsa-miR-4653-3p	1		-0.391	0.5151		
hsa-miR-4655-3p	1		-0.06991	0.9111		
hsa-miR-4668-5p	1		0.3935	0.5123		
hsa-miR-4669	1	1	0.7985	0.1052	0.165	0.7909
hsa-miR-4672	1	1	0.8227	0.0872	0.7563	0.139
hsa-miR-4687-3p	1	1	0.9638	0.0082	0.9709	0.0059
hsa-miR-4689		1			-0.07152	0.909
hsa-miR-4701-3p	1		-0.2466	0.6892		
hsa-miR-4701-5p	1		-0.5948	0.2901		
hsa-miR-4713-3p	1		-0.6681	0.2177		
hsa-miR-4716-3p	1		-0.1074	0.8636		
hsa-miR-4721	1		0.8286	0.0829		
hsa-miR-4728-5p	1		0.7437	0.1497		
hsa-miR-4739	1		0.9954	0.0004		
hsa-miR-4741	1	1	0.7295	0.1618	0.7736	0.1248
hsa-miR-4745-5p	1		0.7918	0.1104		
hsa-miR-4746-3p	1		0.7869	0.1142		
hsa-miR-4750-5p	1		0.1704	0.7841		
hsa-miR-4763-3p	1		0.9697	0.0063		
hsa-miR-4767		1			0.8982	0.0384
hsa-miR-4769-3p	1	1	-0.164	0.7921	0.8982	0.0384

SystematicName	int-miR	sec-miR	int-miR Pearson r	int-miR p-value	sec-miR Pearson r	sec-miR p-value
hsa-miR-4787-3p	1	1	0.8982	0.0384	0.9665	0.0073
hsa-miR-4787-5p	1	1	0.9503	0.0132	-0.8274	0.0838
hsa-miR-4788	1		0.981	0.0031		
hsa-miR-483-5p		1			0.8982	0.0384
hsa-miR-484	1		-0.1893	0.7604		
hsa-miR-485-3p	1		-0.8508	0.0676		
hsa-miR-487a-3p	1		-0.3688	0.5413		
hsa-miR-487b-3p	1		-0.1879	0.7621		
hsa-miR-493-5p	1		0.6741	0.2121		
hsa-miR-494-3p	1	1	0.8448	0.0717	0.02533	0.9678
hsa-miR-495-3p	1		-0.6566	0.2287		
hsa-miR-497-5p	1		-0.8221	0.0876		
hsa-miR-5001-5p	1	1	0.945	0.0154	-0.9941	0.0005
hsa-miR-5006-5p	1		0.3762	0.5326		
hsa-miR-503-5p	1		-0.197	0.7508		
hsa-miR-505-3p	1		0.1487	0.8114		
hsa-miR-5088-5p	1		0.04887	0.9378		
hsa-miR-5090	1		-0.4766	0.417		
hsa-miR-5100	1	1	-0.02073	0.9736	-0.816	0.0921
hsa-miR-513a-5p	1		0.2727	0.6571		
hsa-miR-5194	1		-0.2157	0.7275		
hsa-miR-532-3p	1		-0.3849	0.5223		
hsa-miR-532-5p	1		-0.7345	0.1576		
hsa-miR-539-5p	1		0.165	0.7909		
hsa-miR-543	1		-0.4508	0.4462		
hsa-miR-548ae-3p	1		0.2261	0.7146		
hsa-miR-548aj-3p	1		-0.008363	0.9894		
hsa-miR-548ap-3p	1		0.2073	0.7379		
hsa-miR-548c-3p	1		0.3583	0.5537		
hsa-miR-548f-3p	1		0.1333	0.8308		

SystematicName	int-miR	sec-miR	int-miR Pearson r	int-miR p-value	sec-miR Pearson r	sec-miR p-value
hsa-miR-548x-3p	1		0.1864	0.764		
hsa-miR-550b-2-5p		1			0.8982	0.0384
hsa-miR-5581-5p	1		-0.4416	0.4566		
hsa-miR-5703		1			0.8982	0.0384
hsa-miR-572	1	1	0.9637	0.0082	-0.9197	0.027
hsa-miR-5739	1	1	0.1273	0.8384	0.505	0.3855
hsa-miR-574-3p	1		-0.3826	0.525		
hsa-miR-574-5p	1		0.5947	0.2901		
hsa-miR-575	1		0.2285	0.7116		
hsa-miR-5787	1	1	0.9684	0.0067	0.8982	0.0384
hsa-miR-590-5p	1		-0.007571	0.9904		
hsa-miR-6068	1	1	0.9759	0.0045	-0.8987	0.0381
hsa-miR-6076	1		0.7611	0.135		
hsa-miR-6085	1		0.02781	0.9646		
hsa-miR-6087	1	1	0.9646	0.008	0.9087	0.0327
hsa-miR-6088	1	1	0.9778	0.004	0.1533	0.8056
hsa-miR-6089	1	1	0.9867	0.0018	0.6303	0.2543
hsa-miR-6090	1	1	0.994	0.0006	0.8496	0.0684
hsa-miR-6124	1	1	0.3935	0.5123	0.8982	0.0384
hsa-miR-6125	1	1	0.9632	0.0084	-0.4891	0.4031
hsa-miR-6126	1		0.9872	0.0017		
hsa-miR-6127	1	1	-0.791	0.1111	0.8982	0.0384
hsa-miR-6131	1		-0.6325	0.2522		
hsa-miR-6165	1	1	0.01994	0.9746	0.8982	0.0384
hsa-miR-625-5p	1		0.4817	0.4113		
hsa-miR-629-3p	1		0.8982	0.0384		
hsa-miR-630		1			0.984	0.0024
hsa-miR-636		1			0.9822	0.0028
hsa-miR-638	1	1	0.9368	0.0189	-0.9047	0.0348
hsa-miR-642a-3p	1		0.982	0.0029		

SystematicName	int-miR	sec-miR	int-miR Pearson r	int-miR p-value	sec-miR Pearson r	sec-miR p-value
hsa-miR-642b-3p	1		0.9743	0.0049		
hsa-miR-6510-5p		1			0.8982	0.0384
hsa-miR-6512-5p	1		0.009061	0.9885		
hsa-miR-654-3p	1		-0.7684	0.129		
hsa-miR-660-5p	1		-0.3194	0.6004		
hsa-miR-664b-3p	1		0.01181	0.985		
hsa-miR-6717-5p	1		-0.7617	0.1346		
hsa-miR-6723-5p	1		-0.2949	0.63		
hsa-miR-6724-5p	1	1	0.9123	0.0308	0.9759	0.0045
hsa-miR-6727-5p	1		0.8982	0.0384		
hsa-miR-6728-5p	1		0.9978	0.0001		
hsa-miR-6734-5p	1		-0.4206	0.4807		
hsa-miR-6737-3p	1		-0.2217	0.7201		
hsa-miR-6740-5p	1	1	-0.5816	0.3037	0.8982	0.0384
hsa-miR-6749-5p	1	1	0.7909	0.1111	0.977	0.0042
hsa-miR-6756-5p	1		0.7311	0.1605		
hsa-miR-6763-5p	1		0.6217	0.2629		
hsa-miR-6767-5p	1		-0.5691	0.3167		
hsa-miR-6768-5p	1		0.9778	0.004		
hsa-miR-6769b-5p	1		0.6984	0.1896		
hsa-miR-6780a-5p	1		0.25	0.685		
hsa-miR-6780b-5p	1	1	-0.4476	0.4498	0.8982	0.0384
hsa-miR-6785-5p	1		0.9626	0.0086		
hsa-miR-6789-5p	1		0.4499	0.4471		
hsa-miR-6793-5p		1			-0.02889	0.9632
hsa-miR-6800-5p	1	1	0.9653	0.0077	0.877	0.0508
hsa-miR-6803-5p	1	1	0.8728	0.0534	0.9598	0.0096
hsa-miR-6807-5p	1		0.323	0.596		
hsa-miR-6808-5p	1		0.613	0.2716		
hsa-miR-6820-5p	1		-0.0814	0.8965		

SystematicName	int-miR	sec-miR	int-miR Pearson r	int-miR p-value	sec-miR Pearson r	sec-miR p-value
hsa-miR-6821-5p	1	1	0.9768	0.0042	0.9822	0.0028
hsa-miR-6826-5p	1	1	0.3853	0.5219	-0.2209	0.721
hsa-miR-6829-5p	1		0.266	0.6654		
hsa-miR-6834-3p	1		-0.4766	0.417		
hsa-miR-6840-3p	1		0.3126	0.6086		
hsa-miR-6850-5p	1	1	0.955	0.0114	0.8982	0.0384
hsa-miR-6860	1		0.07277	0.9074		
hsa-miR-6869-5p	1	1	0.9806	0.0032	0.8406	0.0745
hsa-miR-6870-5p	1		-0.218	0.7247		
hsa-miR-6875-5p	1	1	-0.8258	0.085	0.8451	0.0714
hsa-miR-6879-5p	1	1	-0.3968	0.5084	0.8982	0.0384
hsa-miR-6887-5p	1		0.5128	0.377		
hsa-miR-6891-5p	1	1	0.9927	0.0007	0.8982	0.0384
hsa-miR-6892-5p		1			0.8982	0.0384
hsa-miR-6893-5p	1		0.9153	0.0292		
hsa-miR-7-5p	1		-0.2014	0.7453		
hsa-miR-7107-5p	1		0.9003	0.0372		
hsa-miR-7108-5p	1	1	0.8982	0.0384	0.8982	0.0384
hsa-miR-7110-5p	1	1	0.7496	0.1446	0.8982	0.0384
hsa-miR-7114-5p	1		0.2513	0.6835		
hsa-miR-7150	1	1	0.9817	0.003	0.9048	0.0348
hsa-miR-758-3p	1		-0.5027	0.388		
hsa-miR-760		1			-0.0823	0.8953
hsa-miR-762	1	1	0.7969	0.1065	0.8982	0.0384
hsa-miR-7641	1	1	-0.5507	0.3361	0.408	0.4954
hsa-miR-769-5p	1		0.8081	0.098		
hsa-miR-7704	1	1	0.966	0.0075	-0.9769	0.0042
hsa-miR-7847-3p	1	1	0.8982	0.0384	0.165	0.7909
hsa-miR-7975	1	1	-0.5039	0.3867	-0.7958	0.1073
hsa-miR-7977	1	1	-0.04004	0.949	-0.7914	0.1107

SystematicName	int-miR	sec-miR	int-miR Pearson r	int-miR p-value	sec-miR Pearson r	sec-miR p-value
hsa-miR-8063	1		-0.1271	0.8386		
hsa-miR-8069	1	1	0.6011	0.2836	-0.7642	0.1325
hsa-miR-8072	1		0.9844	0.0023		
hsa-miR-874-3p	1		0.5759	0.3096		
hsa-miR-92a-3p	1		-0.7077	0.1811		
hsa-miR-93-5p	1		-0.1877	0.7624		
hsa-miR-937-5p	1		0.9639	0.0082		
hsa-miR-939-5p	1	1	0.6596	0.2258	0.9651	0.0078
hsa-miR-98-5p	1		-0.3673	0.543		
hsa-miR-99a-5p	1		-0.02833	0.9639		
hsa-miR-99b-5p	1		-0.6539	0.2313		

Supplementary Table S5. Spearman correlation coefficients and p values for comparisons between endothelium-related miRNA signatures in paired serum (S1-4E) and plasma (P1-4E) samples as well as average HUVEC intracellular (A) and secretory (B) miRNA signatures.

r_S	P1-E	P2-E	P3-E	P4-E	S1-E	S2-E	S3-E	S4-E	A	B
P1-E	1									
P2-E	0.942789	1								
P3-E	0.944612	0.9360566	1							
P4-E	0.881923	0.8349181	0.9341266	1						
S1-E	0.683021	0.7129387	0.6415027	0.4577195	1					
S2-E	0.669599	0.7387881	0.6548413	0.454349	0.915322	1				
S3-E	0.690023	0.7270362	0.6654546	0.517137	0.9108559	0.8782012	1			
S4-E	0.771457	0.8042147	0.7418416	0.6016612	0.8861202	0.8875226	0.9089665	1		
A	0.467414	0.4244222	0.4933671	0.6109889	0.0877226	0.11349	0.193544	0.2408302	1	
B	0.485579	0.5710907	0.4792628	0.2632688	0.7624915	0.8043557	0.7243951	0.7058965	0.015345	1

p	P1-E	P2-E	P3-E	P4-E	S1-E	S2-E	S3-E	S4-E	A	B
P1-E		Approximate	Approximate	Approximate	Approximate	Approximate	Approximate	Approximate	Approximate	Approximate
P2-E	2.4E-194		Approximate	Approximate	Approximate	Approximate	Approximate	Approximate	Approximate	Approximate
P3-E	4.2E-197	6.45E-185		Approximate	Approximate	Approximate	Approximate	Approximate	Approximate	Approximate
P4-E	1.1E-133	1.45E-106	2.11E-182		Approximate	Approximate	Approximate	Approximate	Approximate	Approximate
S1-E	6E-57	4.225E-64	2.457E-48	2.296E-22		Approximate	Approximate	Approximate	Approximate	Approximate
S2-E	5.19E-54	4.607E-71	5.911E-51	5.055E-22	2.85E-161		Approximate	Approximate	Approximate	Approximate
S3-E	1.52E-58	8.471E-68	3.9E-53	4.462E-29	5.63E-157	3.73E-131		Approximate	Approximate	Approximate
S4-E	3.82E-81	4.254E-93	6.112E-72	3.088E-41	1.13E-136	1.08E-137	3.16E-155		Approximate	Approximate
A	2.26E-23	3.852E-19	3.111E-26	8.274E-43	0.0778451	0.0223559	8.852E-05	9.391E-07		Approximate
B	2.38E-25	1.978E-36	1.196E-24	7.576E-08	3.236E-78	3.737E-93	4.347E-67	2.457E-62	0.7581768	

Supplementary Table S6. HEX codes used to signify each individual miRNA on Figures throughout the paper.

miRNA	HEX	miRNA	HEX	miRNA	HEX	miRNA	HEX	miRNA	HEX	miRNA	HEX
hsa-let-7a-5p	#9875a4	hsa-miR-1185-2-3p	#d48ea7	hsa-miR-125a-3p	#935b45	hsa-miR-1288-3p	#3f4310	hsa-miR-136-3p	#99a9e4	hsa-miR-1537-3p	#d996c4
hsa-let-7b-3p	#6e5227	hsa-miR-1202	#5f90c0	hsa-miR-125a-5p	#515522	hsa-miR-1288-5p	#9fa36b	hsa-miR-136-5p	#68262a	hsa-miR-1538	#0184b1
hsa-let-7b-5p	#5db19f	hsa-miR-1207-5p	#7e4f32	hsa-miR-125b-2-3p	#763a33	hsa-miR-129-1-3p	#a96d90	hsa-miR-137	#8cb682	hsa-miR-1539	#006243
hsa-let-7c-5p	#b192c4	hsa-miR-1208	#a57aa7	hsa-miR-125b-5p	#504979	hsa-miR-129-2-3p	#537547	hsa-miR-138-5p	#5f274d	hsa-miR-154-3p	#b3ad6c
hsa-let-7d-3p	#7c405c	hsa-miR-122-5p	#443461	hsa-miR-126-3p	#937d4b	hsa-miR-1290	#98648c	hsa-miR-139-3p	#005c8b	hsa-miR-154-5p	#005488
hsa-let-7d-5p	#ba7d9f	hsa-miR-1224-3p	#7a3c55	hsa-miR-1260b	#70804c	hsa-miR-1291	#a898ce	hsa-miR-139-5p	#78b990	hsa-miR-155-5p	#018eb8
hsa-let-7e-5p	#704e27	hsa-miR-1224-5p	#7b4466	hsa-miR-1261	#77b591	hsa-miR-1295a	#9f80b2	hsa-miR-140-3p	#db9d7b	hsa-miR-1587	#6b222d
hsa-let-7f-1-3p	#b5706d	hsa-miR-1225-5p	#663520	hsa-miR-1266-3p	#6d996d	hsa-miR-1296-3p	#20441b	hsa-miR-140-5p	#113c6d	hsa-miR-15a-3p	#6b2132
hsa-let-7f-5p	#d08886	hsa-miR-1226-5p	#bf798e	hsa-miR-1267	#a7616d	hsa-miR-1296-5p	#675b8d	hsa-miR-141-3p	#2e420d	hsa-miR-15a-5p	#56bca6
hsa-let-7g-3p	#c47f77	hsa-miR-1227-3p	#c0787e	hsa-miR-1268a	#c67d88	hsa-miR-1299	#5cbaaf	hsa-miR-142-3p	#502e5d	hsa-miR-15b-3p	#df94bc
hsa-let-7g-5p	#4e4413	hsa-miR-1227-5p	#854e71	hsa-miR-1268b	#9080b4	hsa-miR-1301-3p	#004a76	hsa-miR-142-5p	#57b5df	hsa-miR-15b-5p	#006c9f
hsa-let-7i-3p	#be8eba	hsa-miR-1228-3p	#d08d7d	hsa-miR-127-3p	#7899cd	hsa-miR-1301-5p	#9ea8e0	hsa-miR-144-3p	#004c7d	hsa-miR-16-1-3p	#0096be
hsa-let-7i-5p	#6a3b61	hsa-miR-1228-5p	#4d3d6b	hsa-miR-1270	#5b4371	hsa-miR-1304-3p	#e39798	hsa-miR-144-5p	#006b53	hsa-miR-16-2-3p	#00461e
hsa-miR-1-3p	#d88fa0	hsa-miR-1229-3p	#613962	hsa-miR-1271-5p	#dc97b4	hsa-miR-1304-5p	#e0998b	hsa-miR-145-5p	#e7949d	hsa-miR-16-5p	#572a5b
hsa-miR-100-5p	#a96883	hsa-miR-1229-5p	#652c25	hsa-miR-1273c	#9b754b	hsa-miR-1305	#70b99b	hsa-miR-146a-5p	#e5978a	hsa-miR-17-3p	#213a70
hsa-miR-101-3p	#7d3c4a	hsa-miR-1233-3p	#c18c6b	hsa-miR-1273d	#3e6393	hsa-miR-1306-3p	#4a3a08	hsa-miR-146b-5p	#14451b	hsa-miR-17-5p	#612d10
hsa-miR-101-5p	#635927	hsa-miR-1233-5p	#49825f	hsa-miR-1273e	#516395	hsa-miR-1306-5p	#8dace3	hsa-miR-1470	#29430f	hsa-miR-181a-2-3p	#424000
hsa-miR-103a-2-5p	#925b7f	hsa-miR-1236-3p	#b8a16d	hsa-miR-1273f	#4d6130	hsa-miR-1307-3p	#007568	hsa-miR-1471	#0084ad	hsa-miR-181a-3p	#25430c
hsa-miR-103a-3p	#d391b0	hsa-miR-1236-5p	#4a325e	hsa-miR-1273g-3p	#b16c82	hsa-miR-1307-5p	#4e3809	hsa-miR-148a-3p	#c99bd1	hsa-miR-181a-5p	#004f29
hsa-miR-106a-3p	#8c4c64	hsa-miR-1237-3p	#8d4b4d	hsa-miR-1273g-5p	#a88658	hsa-miR-130a-3p	#56340e	hsa-miR-148a-5p	#66281e	hsa-miR-181b-2-3p	#86aceb
hsa-miR-106b-3p	#7e6899	hsa-miR-1238-3p	#a67054	hsa-miR-1273h-3p	#774c76	hsa-miR-130b-3p	#b8a1d8	hsa-miR-148b-3p	#c0a96d	hsa-miR-181b-5p	#e59880
hsa-miR-106b-5p	#5b2e54	hsa-miR-1238-5p	#663153	hsa-miR-1273h-5p	#8f5344	hsa-miR-132-3p	#5a3211	hsa-miR-148b-5p	#e89592	hsa-miR-181c-3p	#423268
hsa-miR-107	#9e5a5d	hsa-miR-1246	#7278ac	hsa-miR-1275	#c39a6e	hsa-miR-132-5p	#e395a4	hsa-miR-149-3p	#01a1b4	hsa-miR-181c-5p	#e792af
hsa-miR-10a-3p	#8f7445	hsa-miR-1249-3p	#83414b	hsa-miR-1278	#829e6b	hsa-miR-1321	#393f09	hsa-miR-149-5p	#68223f	hsa-miR-181d-5p	#49b6e3
hsa-miR-10a-5p	#d28d81	hsa-miR-1249-5p	#ac705b	hsa-miR-128-1-5p	#6f3034	hsa-miR-1322	#0094a5	hsa-miR-150-3p	#e294b5	hsa-miR-182-3p	#82b882
hsa-miR-10b-3p	#652a45	hsa-miR-1250-3p	#b06a7b	hsa-miR-128-3p	#a9759e	hsa-miR-133a-3p	#074522	hsa-miR-150-5p	#e693aa	hsa-miR-182-5p	#57b4e7
hsa-miR-10b-5p	#5a4978	hsa-miR-1250-5p	#866139	hsa-miR-1281	#a6945f	hsa-miR-133b	#da97bd	hsa-miR-151a-3p	#c39dd7	hsa-miR-1825	#5f2654
hsa-miR-1180-3p	#715786	hsa-miR-1251-3p	#4a5687	hsa-miR-1284	#22416f	hsa-miR-134-3p	#cfa374	hsa-miR-151a-5p	#d99e75	hsa-miR-1827	#0178ab
hsa-miR-1181	#884b41	hsa-miR-1253	#9d5c76	hsa-miR-1285-3p	#bb82a9	hsa-miR-134-5p	#67243a	hsa-miR-151b	#64244a	hsa-miR-183-3p	#642a14
hsa-miR-1182	#855b86	hsa-miR-1254	#c5819c	hsa-miR-1286	#5d689b	hsa-miR-1343-3p	#632b1c	hsa-miR-152-3p	#d498ca	hsa-miR-183-5p	#b1a2e3
hsa-miR-1183	#cc9b75	hsa-miR-1255a	#336d9a	hsa-miR-1287-3p	#89a0d6	hsa-miR-1343-5p	#552c58	hsa-miR-152-5p	#9ab375	hsa-miR-184	#c9a66a
hsa-miR-1185-1-3p	#ca92ba	hsa-miR-1255b-5p	#92ad79	hsa-miR-1287-5p	#4d4f80	hsa-miR-135a-3p	#017665	hsa-miR-153-3p	#543503	hsa-miR-185-5p	#01a4c7

miRNA	HEX	miRNA	HEX	miRNA	HEX	miRNA	HEX	miRNA	HEX	miRNA	HEX
hsa-miR-186-3p	#6c1f38	hsa-miR-1972	#00a8d2	hsa-miR-212-3p	#006d46	hsa-miR-2681-5p	#dc9e67	hsa-miR-30c-2-3p	#006534	hsa-miR-3150a-5p	#00612c
hsa-miR-186-5p	#01a6a5	hsa-miR-1973	#a7b16a	hsa-miR-214-3p	#ee8eab	hsa-miR-26a-5p	#004e8e	hsa-miR-30c-5p	#019ada	hsa-miR-3150b-5p	#ef9373
hsa-miR-187-5p	#b9ab68	hsa-miR-1976	#e591ba	hsa-miR-215-5p	#2d3673	hsa-miR-26b-5p	#aab062	hsa-miR-30d-3p	#9eb363	hsa-miR-3151-3p	#685400
hsa-miR-188-3p	#bb9ede	hsa-miR-198	#02a4d1	hsa-miR-216a-5p	#c9a662	hsa-miR-27a-3p	#4dbe9a	hsa-miR-30d-5p	#684d00	hsa-miR-3152-3p	#f3917a
hsa-miR-188-5p	#533c00	hsa-miR-199a-3p	#004d21	hsa-miR-217	#014582	hsa-miR-27b-3p	#d4a262	hsa-miR-30e-3p	#67bc84	hsa-miR-3154	#80b971
hsa-miR-18a-5p	#69261c	hsa-miR-199a-5p	#00643e	hsa-miR-218-5p	#a4a4ed	hsa-miR-28-3p	#004711	hsa-miR-30e-5p	#e191ce	hsa-miR-3156-5p	#008e65
hsa-miR-18b-3p	#008670	hsa-miR-199b-5p	#006f4c	hsa-miR-219a-2-3p	#3c3370	hsa-miR-28-5p	#505000	hsa-miR-31-3p	#91a8f7	hsa-miR-3157-3p	#562769
hsa-miR-18b-5p	#60b3eb	hsa-miR-19a-3p	#254406	hsa-miR-219a-5p	#e29a6f	hsa-miR-2861	#1cbfb1	hsa-miR-31-5p	#455300	hsa-miR-3157-5p	#00aced
hsa-miR-1908-3p	#2d376f	hsa-miR-19b-1-5p	#01bad7	hsa-miR-219b-5p	#6e1a42	hsa-miR-296-5p	#672808	hsa-miR-3115	#4f2b6c	hsa-miR-3158-5p	#f98a99
hsa-miR-1908-5p	#009180	hsa-miR-19b-2-5p	#45306a	hsa-miR-22-3p	#00a08b	hsa-miR-297	#017348	hsa-miR-3117-3p	#79acf9	hsa-miR-3160-3p	#6f4f00
hsa-miR-1909-5p	#682047	hsa-miR-19b-3p	#ef9095	hsa-miR-22-5p	#0082be	hsa-miR-298	#6d194b	hsa-miR-3120-3p	#00a283	hsa-miR-3160-5p	#ea976a
hsa-miR-190a-3p	#009e94	hsa-miR-200a-3p	#0171ab	hsa-miR-221-3p	#711a2d	hsa-miR-299-3p	#114608	hsa-miR-3121-3p	#f58f84	hsa-miR-3161	#74181d
hsa-miR-190a-5p	#573700	hsa-miR-200b-3p	#00b9bd	hsa-miR-221-5p	#613c00	hsa-miR-299-5p	#00521e	hsa-miR-3124-5p	#d794d8	hsa-miR-3162-3p	#760e3a
hsa-miR-191-3p	#cb9ad6	hsa-miR-200c-3p	#91b673	hsa-miR-222-3p	#01ab9b	hsa-miR-29a-3p	#45b4f4	hsa-miR-3125	#004d14	hsa-miR-3162-5p	#5f2263
hsa-miR-191-5p	#004b83	hsa-miR-202-3p	#b99fe4	hsa-miR-222-5p	#f28e9f	hsa-miR-29b-1-5p	#721a23	hsa-miR-3126-3p	#273679	hsa-miR-3163	#00448a
hsa-miR-1910-5p	#00477f	hsa-miR-204-5p	#2abeb7	hsa-miR-223-3p	#47bea1	hsa-miR-29b-2-5p	#ed8db8	hsa-miR-3127-5p	#6c4300	hsa-miR-3165	#adb05a
hsa-miR-1914-3p	#d3a16b	hsa-miR-205-3p	#02a2d3	hsa-miR-223-5p	#70193e	hsa-miR-29b-3p	#02a6e1	hsa-miR-3128	#6ebb7e	hsa-miR-3170	#c6a857
hsa-miR-1915-3p	#01795c	hsa-miR-206	#ed9385	hsa-miR-224-5p	#7badf4	hsa-miR-29c-3p	#701e1b	hsa-miR-3129-3p	#0075ba	hsa-miR-3171	#e19c61
hsa-miR-192-3p	#005831	hsa-miR-208a-5p	#a0b26a	hsa-miR-2276-3p	#ee8eb0	hsa-miR-29c-5p	#e7986e	hsa-miR-3131	#672b00	hsa-miR-3173-3p	#f889a7
hsa-miR-192-5p	#018cbe	hsa-miR-20a-3p	#00aa9f	hsa-miR-2277-3p	#60235a	hsa-miR-300	#674500	hsa-miR-3132	#6a3500	hsa-miR-3174	#004c93
hsa-miR-193a-3p	#24bada	hsa-miR-20a-5p	#12450f	hsa-miR-2278	#5d4800	hsa-miR-301a-3p	#69aff7	hsa-miR-3135b	#0089cd	hsa-miR-3176	#a6a2f5
hsa-miR-193a-5p	#424400	hsa-miR-20b-5p	#77ba83	hsa-miR-2355-5p	#5ebc90	hsa-miR-301b-3p	#db93d2	hsa-miR-3136-3p	#ec8dc0	hsa-miR-3177-3p	#174900
hsa-miR-193b-3p	#00669e	hsa-miR-21-3p	#008263	hsa-miR-2392	#e79873	hsa-miR-302f	#88b871	hsa-miR-3136-5p	#00b299	hsa-miR-3179	#771029
hsa-miR-193b-5p	#8caaed	hsa-miR-21-5p	#2d4400	hsa-miR-23a-3p	#574c00	hsa-miR-3064-3p	#671d55	hsa-miR-3137	#0180c5	hsa-miR-3180-3p	#635b00
hsa-miR-194-3p	#5d2658	hsa-miR-210-3p	#003b76	hsa-miR-23a-5p	#3a4b00	hsa-miR-3064-5p	#015a9d	hsa-miR-3138	#721345	hsa-miR-3180-5p	#e59a63
hsa-miR-194-5p	#d0a369	hsa-miR-210-5p	#4e4900	hsa-miR-23b-3p	#e391c6	hsa-miR-3074-3p	#423071	hsa-miR-3140-3p	#026bb1	hsa-miR-3183	#ca98e6
hsa-miR-195-3p	#007db3	hsa-miR-211-3p	#01b1dd	hsa-miR-23b-5p	#6b2413	hsa-miR-3074-5p	#2b4a00	hsa-miR-3141	#651e5b	hsa-miR-3184-3p	#f98c86
hsa-miR-195-5p	#642250	hsa-miR-211-5p	#632c08	hsa-miR-24-1-5p	#614600	hsa-miR-30a-3p	#51b3f7	hsa-miR-3145-5p	#425400	hsa-miR-3186-3p	#ef946e
hsa-miR-196a-5p	#6e1d31	hsa-miR-2110	#01b7b4	hsa-miR-24-3p	#009578	hsa-miR-30a-5p	#6b1a50	hsa-miR-3146	#5c5700	hsa-miR-3187-3p	#00ae8d
hsa-miR-196b-3p	#d497d0	hsa-miR-2113	#00619e	hsa-miR-2467-3p	#721a29	hsa-miR-30b-3p	#f68d90	hsa-miR-3147	#00a5e6	hsa-miR-3188	#1e4c00
hsa-miR-196b-5p	#99a7ec	hsa-miR-2116-3p	#512b65	hsa-miR-25-3p	#016bab	hsa-miR-30b-5p	#f58ba2	hsa-miR-3148	#98a6f7	hsa-miR-3189-3p	#57b1fe
hsa-miR-197-5p	#ed928d	hsa-miR-2117	#5f3400	hsa-miR-25-5p	#b7ac5f	hsa-miR-30c-1-3p	#c59ae4	hsa-miR-3149	#009d7a	hsa-miR-3190-3p	#019d75

miRNA	HEX	miRNA	HEX	miRNA	HEX	miRNA	HEX	miRNA	HEX	miRNA	HEX
hsa-miR-3190-5p	#ef8abf	hsa-miR-335-5p	#e28fd5	hsa-miR-3617-5p	#696500	hsa-miR-3679-5p	#456200	hsa-miR-377-5p	#68bd71	hsa-miR-3940-3p	#7b0053
hsa-miR-3191-5p	#064600	hsa-miR-337-3p	#b19ff5	hsa-miR-362-3p	#fb8c7a	hsa-miR-3680-3p	#472b78	hsa-miR-378a-3p	#0090e5	hsa-miR-3940-5p	#253385
hsa-miR-3192-3p	#711e10	hsa-miR-337-5p	#85a9ff	hsa-miR-362-5p	#770346	hsa-miR-3681-3p	#76024c	hsa-miR-378a-5p	#f885c0	hsa-miR-3942-5p	#ff837a
hsa-miR-3192-5p	#f688b0	hsa-miR-338-3p	#00662b	hsa-miR-3620-3p	#e68dd4	hsa-miR-3682-3p	#815f00	hsa-miR-378b	#0160b4	hsa-miR-3944-5p	#87002e
hsa-miR-3194-5p	#1b377e	hsa-miR-338-5p	#3f2f78	hsa-miR-3620-5p	#baac4f	hsa-miR-3683	#5d6800	hsa-miR-378c	#542574	hsa-miR-3945	#7d2b00
hsa-miR-3195	#754600	hsa-miR-339-3p	#0175c2	hsa-miR-3621	#722800	hsa-miR-3685	#ef9561	hsa-miR-378d	#741a03	hsa-miR-3960	#82ba5e
hsa-miR-3196	#5fb0ff	hsa-miR-339-5p	#003a83	hsa-miR-3622a-3p	#cb96ea	hsa-miR-3688-3p	#245700	hsa-miR-378e	#007f40	hsa-miR-3976	#576e00
hsa-miR-3197	#703300	hsa-miR-33a-3p	#d8a056	hsa-miR-3622b-5p	#5abe7f	hsa-miR-3689a-3p	#00b68b	hsa-miR-378f	#3ec087	hsa-miR-409-3p	#6f7200
hsa-miR-3198	#7babfe	hsa-miR-33a-5p	#6cbc77	hsa-miR-363-3p	#763100	hsa-miR-3689a-5p	#00601c	hsa-miR-378g	#9fa0ff	hsa-miR-409-5p	#ef9559
hsa-miR-3199	#008352	hsa-miR-33b-3p	#0183d0	hsa-miR-363-5p	#fe8983	hsa-miR-3689b-3p	#de9e53	hsa-miR-378i	#0159ad	hsa-miR-410-3p	#008949
hsa-miR-32-3p	#0198e1	hsa-miR-33b-5p	#f68f74	hsa-miR-3646	#546400	hsa-miR-3689d	#ff848a	hsa-miR-379-5p	#ff7e99	hsa-miR-411-3p	#ed88d4
hsa-miR-32-5p	#6c2402	hsa-miR-340-3p	#763a00	hsa-miR-3648	#710d54	hsa-miR-3689f	#f387c5	hsa-miR-380-5p	#ff7e94	hsa-miR-411-5p	#880049
hsa-miR-3200-3p	#004b08	hsa-miR-340-5p	#7a062e	hsa-miR-3650	#8aa5ff	hsa-miR-369-5p	#5e1f6b	hsa-miR-381-3p	#d592e9	hsa-miR-412-5p	#f98f65
hsa-miR-3200-5p	#0267b2	hsa-miR-342-3p	#7a5600	hsa-miR-3651	#7c003b	hsa-miR-3691-3p	#0182d5	hsa-miR-382-5p	#78a1ff	hsa-miR-421	#8d6700
hsa-miR-3202	#fc8992	hsa-miR-342-5p	#014590	hsa-miR-3652	#dc90de	hsa-miR-3691-5p	#7d0045	hsa-miR-383-5p	#7d3000	hsa-miR-422a	#fe82ba
hsa-miR-320a	#00a3eb	hsa-miR-345-3p	#02b5fe	hsa-miR-3653-3p	#e49b59	hsa-miR-3692-5p	#006bbe	hsa-miR-3907	#4d6900	hsa-miR-423-3p	#baac45
hsa-miR-320b	#018d5e	hsa-miR-345-5p	#007036	hsa-miR-3653-5p	#0153a2	hsa-miR-370-3p	#cfa54c	hsa-miR-3911	#884900	hsa-miR-423-5p	#b39cfd
hsa-miR-320c	#6b1758	hsa-miR-346	#c09aef	hsa-miR-3654	#f5906c	hsa-miR-3714	#2babff	hsa-miR-3912-3p	#00a0f6	hsa-miR-424-3p	#830023
hsa-miR-320d	#9ea4fa	hsa-miR-34a-5p	#ea8cce	hsa-miR-3656	#7abb6b	hsa-miR-371a-3p	#00abfd	hsa-miR-3913-5p	#003a8b	hsa-miR-424-5p	#847000
hsa-miR-320e	#35c098	hsa-miR-34b-3p	#77121a	hsa-miR-3659	#a1b355	hsa-miR-371a-5p	#eb975b	hsa-miR-3914	#7a0d13	hsa-miR-425-3p	#905d00
hsa-miR-323a-3p	#760942	hsa-miR-34b-5p	#fe869a	hsa-miR-365a-3p	#00854d	hsa-miR-371b-5p	#75170a	hsa-miR-3916	#4ea4ff	hsa-miR-425-5p	#dd8fe6
hsa-miR-324-3p	#015aa5	hsa-miR-3591-3p	#01b792	hsa-miR-365a-5p	#d493e5	hsa-miR-373-5p	#0274c8	hsa-miR-3917	#7abb64	hsa-miR-4252	#a29dff
hsa-miR-324-5p	#ed8bc6	hsa-miR-3605-3p	#d1a452	hsa-miR-3663-3p	#804800	hsa-miR-374a-3p	#007130	hsa-miR-3922-5p	#8db859	hsa-miR-4253	#7b2400
hsa-miR-326	#007640	hsa-miR-3605-5p	#761516	hsa-miR-3663-5p	#761611	hsa-miR-374a-5p	#6f0e5b	hsa-miR-3923	#01b17e	hsa-miR-4254	#442b7e
hsa-miR-328-3p	#00591b	hsa-miR-3609	#790b22	hsa-miR-3664-3p	#67a7ff	hsa-miR-374b-3p	#355f00	hsa-miR-3924	#005605	hsa-miR-4257	#8b0044
hsa-miR-328-5p	#fc898c	hsa-miR-361-3p	#ba9cf3	hsa-miR-3665	#adb050	hsa-miR-374b-5p	#fc84b7	hsa-miR-3926	#e28ddf	hsa-miR-4258	#ff7a98
hsa-miR-329-3p	#606000	hsa-miR-361-5p	#00c09e	hsa-miR-3666	#7c0129	hsa-miR-374c-5p	#824000	hsa-miR-3928-5p	#e59b52	hsa-miR-4259	#8c003b
hsa-miR-330-3p	#78bb71	hsa-miR-3610	#263580	hsa-miR-3667-5p	#ff84a5	hsa-miR-376a-3p	#7b6a00	hsa-miR-3934-5p	#00641b	hsa-miR-4261	#40c081
hsa-miR-330-5p	#395800	hsa-miR-3611	#195000	hsa-miR-3675-3p	#23c196	hsa-miR-376a-5p	#a6a0fe	hsa-miR-3935	#8c6000	hsa-miR-4269	#ff7ba3
hsa-miR-331-3p	#755900	hsa-miR-3613-3p	#bfaa4f	hsa-miR-3677-3p	#019c69	hsa-miR-376b-3p	#014596	hsa-miR-3937	#e98ad8	hsa-miR-4270	#621a6d
hsa-miR-331-5p	#790632	hsa-miR-3614-5p	#fa86b2	hsa-miR-3678-3p	#f886bc	hsa-miR-376c-3p	#885500	hsa-miR-3938	#8c5100	hsa-miR-4271	#57a0ff
hsa-miR-335-3p	#5d2268	hsa-miR-3617-3p	#731b0c	hsa-miR-3679-3p	#00a97a	hsa-miR-377-3p	#21c192	hsa-miR-3939	#667000	hsa-miR-4272	#0276d0

miRNA	HEX	miRNA	HEX	miRNA	HEX	miRNA	HEX	miRNA	HEX	miRNA	HEX
hsa-miR-4274	#009c5f	hsa-miR-4417	#572177	hsa-miR-4466	#ed964e	hsa-miR-4504	#00bf82	hsa-miR-4637	#587c00	hsa-miR-4674	#ff875b
hsa-miR-4281	#dc9f49	hsa-miR-4419a	#3d6b00	hsa-miR-4468	#0165c3	hsa-miR-4505	#ff8068	hsa-miR-4638-3p	#6a8100	hsa-miR-4676-3p	#0060c4
hsa-miR-4282	#00a3fd	hsa-miR-4419b	#667700	hsa-miR-4470	#33c17f	hsa-miR-4507	#954200	hsa-miR-4639-5p	#4e7900	hsa-miR-4676-5p	#aa92ff
hsa-miR-4284	#7e7400	hsa-miR-4422	#975b00	hsa-miR-4472	#8c0059	hsa-miR-4508	#7f000b	hsa-miR-4640-3p	#d3a439	hsa-miR-4677-3p	#432887
hsa-miR-4286	#6e0b61	hsa-miR-4423-3p	#0f358b	hsa-miR-4474-3p	#80ba56	hsa-miR-450a-1-3p	#937e00	hsa-miR-4640-5p	#8d0063	hsa-miR-4685-3p	#758700
hsa-miR-4290	#791c00	hsa-miR-4426	#00b87f	hsa-miR-4474-5p	#950034	hsa-miR-450a-5p	#0193f6	hsa-miR-4644	#ff7077	hsa-miR-4685-5p	#f29448
hsa-miR-4291	#0095f0	hsa-miR-4428	#f784ce	hsa-miR-4476	#ff75a7	hsa-miR-4510	#8d8000	hsa-miR-4646-3p	#ec9846	hsa-miR-4687-3p	#9e4500
hsa-miR-4294	#ff798b	hsa-miR-4429	#82005a	hsa-miR-4477a	#6f0565	hsa-miR-4511	#a07100	hsa-miR-4646-5p	#2698ff	hsa-miR-4687-5p	#a60049
hsa-miR-4297	#ff7d7d	hsa-miR-4430	#e39c49	hsa-miR-4478	#007ada	hsa-miR-4512	#004eac	hsa-miR-4647	#ff746f	hsa-miR-4688	#276f00
hsa-miR-4298	#ce94f2	hsa-miR-4433a-3p	#0088e6	hsa-miR-448	#950051	hsa-miR-4513	#007c2c	hsa-miR-4648	#b1b039	hsa-miR-4689	#ef85e3
hsa-miR-4299	#5e1d71	hsa-miR-4433b-3p	#ea89de	hsa-miR-4481	#e28be7	hsa-miR-4514	#c296fe	hsa-miR-4649-3p	#ff70a9	hsa-miR-4690-5p	#a10034
hsa-miR-4304	#8d4400	hsa-miR-4435	#326800	hsa-miR-4482-5p	#6099ff	hsa-miR-4515	#e89a46	hsa-miR-4651	#003895	hsa-miR-4693-5p	#38c274
hsa-miR-4305	#800019	hsa-miR-4436b-3p	#018c46	hsa-miR-4483	#0053b0	hsa-miR-4516	#ff796e	hsa-miR-4652-3p	#f6924f	hsa-miR-4694-3p	#651175
hsa-miR-4306	#73015c	hsa-miR-4436b-5p	#8b0025	hsa-miR-4484	#c0ab3c	hsa-miR-4518	#9c003e	hsa-miR-4653-3p	#00984c	hsa-miR-4695-3p	#cca733
hsa-miR-431-5p	#c098fa	hsa-miR-4440	#976d00	hsa-miR-4485-3p	#990043	hsa-miR-451a	#9e7700	hsa-miR-4655-3p	#e99943	hsa-miR-4695-5p	#2595ff
hsa-miR-4311	#d1a443	hsa-miR-4441	#003f97	hsa-miR-4485-5p	#a9b241	hsa-miR-451b	#015bbb	hsa-miR-4655-5p	#930021	hsa-miR-4697-3p	#038ef7
hsa-miR-4312	#a5b348	hsa-miR-4442	#3a2d84	hsa-miR-4486	#fb8e5c	hsa-miR-4521	#852100	hsa-miR-4656	#a07f00	hsa-miR-4697-5p	#a3005a
hsa-miR-4313	#d292f0	hsa-miR-4443	#ff759e	hsa-miR-4487	#8d3600	hsa-miR-4522	#892900	hsa-miR-4659a-3p	#7a93ff	hsa-miR-4698	#8e0015
hsa-miR-4314	#849bff	hsa-miR-4444	#006c1c	hsa-miR-4488	#0270d0	hsa-miR-4526	#7b0065	hsa-miR-4659a-5p	#bfab35	hsa-miR-4700-3p	#ab6400
hsa-miR-4317	#01439a	hsa-miR-4445-3p	#8c7900	hsa-miR-4493	#6c0a69	hsa-miR-4530	#007bde	hsa-miR-4659b-3p	#0c6600	hsa-miR-4701-3p	#d390fa
hsa-miR-4318	#0d5b00	hsa-miR-4446-5p	#246400	hsa-miR-4494	#3f7000	hsa-miR-4532	#28308b	hsa-miR-4659b-5p	#007e2a	hsa-miR-4701-5p	#571e7f
hsa-miR-4319	#914a00	hsa-miR-4447	#90b74e	hsa-miR-4495	#ff7677	hsa-miR-4534	#9b0053	hsa-miR-466	#a3004c	hsa-miR-4707-3p	#006dd4
hsa-miR-432-3p	#b4ae42	hsa-miR-4449	#007325	hsa-miR-4496	#00a764	hsa-miR-4535	#ff8462	hsa-miR-4660	#ff6b82	hsa-miR-4707-5p	#d7a235
hsa-miR-432-5p	#0059b3	hsa-miR-4450	#7c7c00	hsa-miR-4497	#68106d	hsa-miR-4538	#6296ff	hsa-miR-4664-3p	#df8cf0	hsa-miR-4710	#b591ff
hsa-miR-4320	#917000	hsa-miR-4451	#7f005e	hsa-miR-4499	#f89057	hsa-miR-4539	#91005f	hsa-miR-4664-5p	#a55a00	hsa-miR-4711-3p	#01ac61
hsa-miR-4322	#691269	hsa-miR-4455	#ff7fc0	hsa-miR-449a	#9f6100	hsa-miR-454-3p	#8d2f00	hsa-miR-4665-5p	#ff6a90	hsa-miR-4713-3p	#a34c00
hsa-miR-4323	#75bc61	hsa-miR-4456	#4a277f	hsa-miR-449b-3p	#412a84	hsa-miR-454-5p	#ff7abf	hsa-miR-4667-3p	#86ba4c	hsa-miR-4713-5p	#868b00
hsa-miR-4324	#ef87d7	hsa-miR-4458	#00a15f	hsa-miR-449b-5p	#ff7183	hsa-miR-455-3p	#9e94ff	hsa-miR-4667-5p	#871c00	hsa-miR-4714-3p	#f582dd
hsa-miR-4326	#7a0c08	hsa-miR-4459	#960049	hsa-miR-449c-3p	#d8a13e	hsa-miR-4632-5p	#ff6d8a	hsa-miR-4668-5p	#870068	hsa-miR-4714-5p	#0074dc
hsa-miR-4327	#85001e	hsa-miR-4462	#8b98ff	hsa-miR-449c-5p	#7c0d00	hsa-miR-4633-5p	#95005d	hsa-miR-4669	#9b002c	hsa-miR-4716-3p	#7c006d
hsa-miR-4329	#004ea7	hsa-miR-4463	#009afa	hsa-miR-4500	#53c06f	hsa-miR-4634	#008a3c	hsa-miR-4672	#a97200	hsa-miR-4716-5p	#ff7763
hsa-miR-433-3p	#873400	hsa-miR-4465	#900055	hsa-miR-4502	#013791	hsa-miR-4635	#a56d00	hsa-miR-4673	#6793ff	hsa-miR-4717-3p	#003c9d

miRNA	HEX	miRNA	HEX	miRNA	HEX	miRNA	HEX	miRNA	HEX	miRNA	HEX
hsa-miR-4717-5p	#818b00	hsa-miR-4748	#f99049	hsa-miR-4792	#9f3500	hsa-miR-5003-5p	#be7300	hsa-miR-514b-5p	#ad4000	hsa-miR-548ae-3p	#ee82f3
hsa-miR-4718	#71bd55	hsa-miR-4749-3p	#758d00	hsa-miR-4793-3p	#ff5e8a	hsa-miR-5006-5p	#3d8bff	hsa-miR-515-5p	#ff665c	hsa-miR-548aj-3p	#ff6ccb
hsa-miR-4719	#4d2384	hsa-miR-4749-5p	#698a00	hsa-miR-4793-5p	#fb7edd	hsa-miR-5007-5p	#65bf52	hsa-miR-516a-5p	#ff5971	hsa-miR-548am-5p	#c40061
hsa-miR-4721	#e489ee	hsa-miR-4750-3p	#b47700	hsa-miR-4796-3p	#972600	hsa-miR-5008-5p	#bf7c00	hsa-miR-5187-5p	#b40033	hsa-miR-548ap-3p	#66037f
hsa-miR-4722-3p	#ff7aca	hsa-miR-4750-5p	#8b8dff	hsa-miR-4797-3p	#b40058	hsa-miR-5009-5p	#5ac058	hsa-miR-5189-3p	#ff7350	hsa-miR-548aq-5p	#4b1f8e
hsa-miR-4723-3p	#213090	hsa-miR-4753-5p	#a7b333	hsa-miR-4800-3p	#8d0005	hsa-miR-500a-3p	#ff6564	hsa-miR-5189-5p	#c38e00	hsa-miR-548au-3p	#9cb626
hsa-miR-4723-5p	#94001b	hsa-miR-4755-3p	#a84c00	hsa-miR-4800-5p	#b58b00	hsa-miR-500a-5p	#521d87	hsa-miR-518e-5p	#8b007d	hsa-miR-548au-5p	#9ca400
hsa-miR-4725-3p	#ff8856	hsa-miR-4756-5p	#006e04	hsa-miR-4802-3p	#930074	hsa-miR-500b-5p	#013198	hsa-miR-5190	#bb5900	hsa-miR-548aw	#0150c2
hsa-miR-4725-5p	#00923f	hsa-miR-4758-3p	#ae5900	hsa-miR-483-3p	#9b0072	hsa-miR-501-3p	#5c1582	hsa-miR-5193	#027df2	hsa-miR-548ay-5p	#c0ab14
hsa-miR-4726-3p	#ff75c1	hsa-miR-4758-5p	#a38dff	hsa-miR-483-5p	#397e00	hsa-miR-501-5p	#e288f8	hsa-miR-5194	#c26a00	hsa-miR-548bb-5p	#acb11a
hsa-miR-4726-5p	#f19544	hsa-miR-4762-5p	#979100	hsa-miR-484	#960012	hsa-miR-5010-3p	#b0006d	hsa-miR-5195-3p	#cda61c	hsa-miR-548c-3p	#c36000
hsa-miR-4727-3p	#880009	hsa-miR-4763-3p	#ac003d	hsa-miR-485-3p	#0056c1	hsa-miR-5010-5p	#005fce	hsa-miR-5195-5p	#e19e26	hsa-miR-548d-3p	#008312
hsa-miR-4728-3p	#990068	hsa-miR-4763-5p	#8a0e00	hsa-miR-485-5p	#a8006c	hsa-miR-5011-5p	#008825	hsa-miR-5196-3p	#b10074	hsa-miR-548d-5p	#ff5088
hsa-miR-4728-5p	#828fff	hsa-miR-4764-3p	#ff69ae	hsa-miR-486-3p	#ed9738	hsa-miR-502-3p	#c18500	hsa-miR-5196-5p	#ff5693	hsa-miR-548e-3p	#00319e
hsa-miR-4730	#a20062	hsa-miR-4767	#ff6184	hsa-miR-486-5p	#c48cff	hsa-miR-502-5p	#b7003f	hsa-miR-5197-3p	#819c00	hsa-miR-548e-5p	#a20011
hsa-miR-4731-3p	#86006e	hsa-miR-4768-3p	#ff8154	hsa-miR-487a-3p	#0075e4	hsa-miR-503-5p	#0042ad	hsa-miR-519b-3p	#950004	hsa-miR-548f-3p	#c7005c
hsa-miR-4732-3p	#ff6582	hsa-miR-4769-3p	#b8ae2c	hsa-miR-487b-3p	#5d8b00	hsa-miR-504-3p	#bc0051	hsa-miR-519d-3p	#4387ff	hsa-miR-548i	#ada500
hsa-miR-4732-5p	#a58900	hsa-miR-4769-5p	#3e7d00	hsa-miR-487b-5p	#2d2c92	hsa-miR-505-3p	#71007a	hsa-miR-520b	#aca000	hsa-miR-548q	#4e9000
hsa-miR-4733-5p	#ff679f	hsa-miR-4774-3p	#ff6474	hsa-miR-491-3p	#7d0077	hsa-miR-505-5p	#a40076	hsa-miR-520e	#d188ff	hsa-miR-548v	#e09f1f
hsa-miR-4734	#621379	hsa-miR-4776-3p	#f79145	hsa-miR-491-5p	#97b735	hsa-miR-508-5p	#01bc67	hsa-miR-525-5p	#b80070	hsa-miR-548w	#dd87ff
hsa-miR-4737	#aa0041	hsa-miR-4776-5p	#fe8d4d	hsa-miR-493-5p	#52c15e	hsa-miR-5088-5p	#920a00	hsa-miR-526b-5p	#6e85ff	hsa-miR-548x-3p	#003baa
hsa-miR-4738-3p	#c9a72e	hsa-miR-4778-5p	#608900	hsa-miR-494-3p	#a60026	hsa-miR-5090	#ff7bdd	hsa-miR-532-3p	#eb992c	hsa-miR-548y	#312997
hsa-miR-4739	#47c16a	hsa-miR-4779	#b1004a	hsa-miR-494-5p	#af9400	hsa-miR-5092	#ff5f68	hsa-miR-532-5p	#0183fa	hsa-miR-550a-3-5p	#c3006d
hsa-miR-4740-3p	#ff7dd2	hsa-miR-4780	#768cff	hsa-miR-495-3p	#f89141	hsa-miR-5093	#a8b327	hsa-miR-539-5p	#9b007e	hsa-miR-550a-3p	#af007d
hsa-miR-4741	#a04000	hsa-miR-4781-3p	#01a04b	hsa-miR-497-5p	#23c36f	hsa-miR-5094	#a987ff	hsa-miR-541-5p	#f79237	hsa-miR-550a-5p	#709c00
hsa-miR-4742-5p	#6e0070	hsa-miR-4782-3p	#006ad6	hsa-miR-498	#972100	hsa-miR-5096	#bc0060	hsa-miR-542-3p	#ff58a1	hsa-miR-550b-2-5p	#c70066
hsa-miR-4743-5p	#007b1e	hsa-miR-4783-3p	#01399e	hsa-miR-4999-3p	#b49200	hsa-miR-5100	#ff5ea7	hsa-miR-542-5p	#3e2592	hsa-miR-551b-3p	#630883
hsa-miR-4745-5p	#bc8fff	hsa-miR-4784	#b35f00	hsa-miR-4999-5p	#989900	hsa-miR-511-3p	#b9ae20	hsa-miR-543	#8aa000	hsa-miR-554	#ba83ff
hsa-miR-4746-3p	#79bc4d	hsa-miR-4785	#ab0034	hsa-miR-499a-5p	#014cb7	hsa-miR-513a-3p	#01b057	hsa-miR-545-3p	#ff764a	hsa-miR-557	#f57eee
hsa-miR-4746-5p	#9d8f00	hsa-miR-4787-3p	#998bff	hsa-miR-5001-5p	#008320	hsa-miR-513a-5p	#ff6bc3	hsa-miR-548a-3p	#820080	hsa-miR-5571-5p	#ff58aa
hsa-miR-4747-3p	#b08300	hsa-miR-4787-5p	#ff5f91	hsa-miR-5002-3p	#b50063	hsa-miR-513b-5p	#4d1f8b	hsa-miR-548aa	#93b82d	hsa-miR-5579-5p	#cb9900
hsa-miR-4747-5p	#ac005a	hsa-miR-4788	#01bc6d	hsa-miR-5003-3p	#bd6f00	hsa-miR-513c-5p	#0185fa	hsa-miR-548ad-5p	#b84d00	hsa-miR-5580-3p	#af82ff

miRNA	HEX	miRNA	HEX	miRNA	HEX	miRNA	HEX	miRNA	HEX	miRNA	HEX
hsa-miR-5581-5p	#ff4f75	hsa-miR-5787	#cd9f00	hsa-miR-6088	#8fb923	hsa-miR-630	#977bff	hsa-miR-6511b-5p	#ff48a7	hsa-miR-6716-5p	#e99300
hsa-miR-5583-3p	#009b34	hsa-miR-579-3p	#d483ff	hsa-miR-6089	#d77c00	hsa-miR-631	#0135ab	hsa-miR-6512-5p	#0168e9	hsa-miR-6717-5p	#561093
hsa-miR-5584-5p	#ff61bd	hsa-miR-579-5p	#cf7000	hsa-miR-609	#ff478e	hsa-miR-633	#54c146	hsa-miR-6513-3p	#e69c02	hsa-miR-6720-3p	#ff4557
hsa-miR-5585-3p	#be0077	hsa-miR-580-3p	#bf0033	hsa-miR-6090	#cf0073	hsa-miR-634	#d70054	hsa-miR-6514-3p	#ce7bff	hsa-miR-6721-5p	#47c245
hsa-miR-5586-3p	#c483ff	hsa-miR-582-3p	#96a900	hsa-miR-610	#009b2c	hsa-miR-636	#c70084	hsa-miR-6515-3p	#a10092	hsa-miR-6722-3p	#ff47ad
hsa-miR-5587-5p	#9281ff	hsa-miR-582-5p	#64bf44	hsa-miR-6124	#389000	hsa-miR-638	#e08d00	hsa-miR-6515-5p	#3f7aff	hsa-miR-6722-5p	#2f25a0
hsa-miR-5588-3p	#c86500	hsa-miR-583	#ad0019	hsa-miR-6125	#cc0042	hsa-miR-640	#8daf00	hsa-miR-6516-3p	#00961a	hsa-miR-6723-5p	#01ac37
hsa-miR-5589-3p	#0144b6	hsa-miR-584-3p	#4881ff	hsa-miR-6126	#c7007e	hsa-miR-641	#87008e	hsa-miR-6516-5p	#00c55b	hsa-miR-6724-5p	#179200
hsa-miR-559	#e485fd	hsa-miR-584-5p	#b63d00	hsa-miR-6127	#a30b00	hsa-miR-642a-3p	#b37bff	hsa-miR-652-3p	#ff683c	hsa-miR-6726-5p	#d2008c
hsa-miR-561-3p	#0172eb	hsa-miR-585-5p	#ff4a78	hsa-miR-6129	#0256d0	hsa-miR-642a-5p	#c5002e	hsa-miR-652-5p	#5c79ff	hsa-miR-6727-3p	#e57800
hsa-miR-564	#a31f00	hsa-miR-589-3p	#daa110	hsa-miR-6130	#a9b306	hsa-miR-642b-3p	#098900	hsa-miR-653-5p	#d00086	hsa-miR-6727-5p	#ff5bce
hsa-miR-566	#5c1188	hsa-miR-590-5p	#43c255	hsa-miR-6131	#be0029	hsa-miR-642b-5p	#67a400	hsa-miR-654-3p	#ff3e94	hsa-miR-6728-3p	#ff5e3d
hsa-miR-568	#009125	hsa-miR-595	#6f0085	hsa-miR-6132	#cb5a00	hsa-miR-643	#ff4e57	hsa-miR-654-5p	#471c98	hsa-miR-6728-5p	#dc0085
hsa-miR-5681b	#ff5cb5	hsa-miR-596	#9f0087	hsa-miR-6133	#5d0c8b	hsa-miR-644a	#70be33	hsa-miR-657	#7ebc23	hsa-miR-6729-3p	#c3001f
hsa-miR-5684	#b94600	hsa-miR-598-3p	#ff6fd9	hsa-miR-6134	#ba0023	hsa-miR-645	#ff4a5c	hsa-miR-659-3p	#ff5847	hsa-miR-6730-3p	#640090
hsa-miR-5685	#d17f00	hsa-miR-598-5p	#ff793f	hsa-miR-615-3p	#ff4ca3	hsa-miR-647	#d26000	hsa-miR-659-5p	#c64300	hsa-miR-6730-5p	#920097
hsa-miR-5690	#ff684d	hsa-miR-601	#d39b00	hsa-miR-616-3p	#c70036	hsa-miR-6499-3p	#ff3e7f	hsa-miR-660-3p	#00bd50	hsa-miR-6731-3p	#ff3962
hsa-miR-5692b	#ff7544	hsa-miR-602	#fa7bed	hsa-miR-616-5p	#0042b9	hsa-miR-6500-3p	#ff4367	hsa-miR-660-5p	#ff435d	hsa-miR-6732-3p	#0032ae
hsa-miR-5695	#80a300	hsa-miR-605-5p	#f19626	hsa-miR-6165	#dc9b00	hsa-miR-6500-5p	#a67aff	hsa-miR-661	#ff6ce2	hsa-miR-6732-5p	#0054d6
hsa-miR-5698	#ff5660	hsa-miR-6068	#b23400	hsa-miR-619-5p	#ff74e6	hsa-miR-6503-3p	#ff56bd	hsa-miR-662	#df0058	hsa-miR-6733-5p	#af0400
hsa-miR-5699-5p	#ff78e4	hsa-miR-6069	#d00062	hsa-miR-621	#f07ffa	hsa-miR-6503-5p	#76aa00	hsa-miR-663a	#b40092	hsa-miR-6734-5p	#fc901c
hsa-miR-570-3p	#ff509b	hsa-miR-607	#93b822	hsa-miR-622	#cc003d	hsa-miR-6504-3p	#84bb22	hsa-miR-663b	#e10062	hsa-miR-6735-3p	#013bb9
hsa-miR-5700	#238500	hsa-miR-6071	#c5a900	hsa-miR-623	#ff7c37	hsa-miR-6505-5p	#dc015c	hsa-miR-664a-3p	#9c0094	hsa-miR-6735-5p	#ff3468
hsa-miR-5703	#a1000a	hsa-miR-6072	#cb81ff	hsa-miR-624-3p	#ff594f	hsa-miR-6506-3p	#78008e	hsa-miR-664a-5p	#f07eff	hsa-miR-6736-3p	#e20082
hsa-miR-5704	#016ce5	hsa-miR-6073	#ff55ae	hsa-miR-624-5p	#ff4594	hsa-miR-6508-5p	#af000d	hsa-miR-664b-3p	#e1006f	hsa-miR-6736-5p	#00b33d
hsa-miR-5708	#b10020	hsa-miR-6074	#00b24d	hsa-miR-625-3p	#9ab611	hsa-miR-6509-3p	#0274f6	hsa-miR-664b-5p	#c2008f	hsa-miR-6737-3p	#8674ff
hsa-miR-572	#d38b00	hsa-miR-6075	#005dd6	hsa-miR-625-5p	#b5008a	hsa-miR-6509-5p	#dd0066	hsa-miR-665	#b878ff	hsa-miR-6737-5p	#56a700
hsa-miR-5739	#c90049	hsa-miR-6076	#242a9c	hsa-miR-627-5p	#c85100	hsa-miR-6510-3p	#ff6143	hsa-miR-668-5p	#950094	hsa-miR-6738-3p	#0148c9
hsa-miR-574-3p	#c9a200	hsa-miR-6083	#402296	hsa-miR-628-3p	#d77000	hsa-miR-6510-5p	#e38a00	hsa-miR-670-5p	#8677ff	hsa-miR-6738-5p	#ee8a00
hsa-miR-574-5p	#bda800	hsa-miR-6085	#717eff	hsa-miR-628-5p	#ff5ec6	hsa-miR-6511a-3p	#459a00	hsa-miR-671-3p	#ea9a02	hsa-miR-6739-3p	#ff2f82
hsa-miR-575	#80bb32	hsa-miR-6086	#017dfb	hsa-miR-629-3p	#af2400	hsa-miR-6511a-5p	#ff3c76	hsa-miR-671-5p	#e57cff	hsa-miR-6739-5p	#016bf2
hsa-miR-576-3p	#5581ff	hsa-miR-6087	#a2ae00	hsa-miR-629-5p	#ff5552	hsa-miR-6511b-3p	#d9004e	hsa-miR-6716-3p	#ff8729	hsa-miR-6740-3p	#4c75ff

miRNA	HEX	miRNA	HEX	miRNA	HEX	miRNA	HEX	miRNA	HEX	miRNA	HEX
hsa-miR-6740-5p	#870098	hsa-miR-6761-3p	#ff841a	hsa-miR-6785-3p	#f50061	hsa-miR-6802-5p	#00ab20	hsa-miR-6830-3p	#d000ab	hsa-miR-6857-3p	#d900b2
hsa-miR-6741-3p	#e7007d	hsa-miR-6762-3p	#ee0058	hsa-miR-6785-5p	#54c22f	hsa-miR-6803-3p	#54079c	hsa-miR-6830-5p	#fa014e	hsa-miR-6857-5p	#ff0ca8
hsa-miR-6741-5p	#1776ff	hsa-miR-6763-3p	#004cd2	hsa-miR-6786-3p	#df71ff	hsa-miR-6803-5p	#5cb500	hsa-miR-6831-3p	#e6002e	hsa-miR-6858-3p	#0026b4
hsa-miR-6743-3p	#ff75f5	hsa-miR-6763-5p	#ff5339	hsa-miR-6786-5p	#ee6d00	hsa-miR-6804-3p	#0046d0	hsa-miR-6831-5p	#66bf00	hsa-miR-6858-5p	#e90029
hsa-miR-6745	#6874ff	hsa-miR-6764-5p	#6c70ff	hsa-miR-6787-3p	#ff245c	hsa-miR-6805-5p	#ff6225	hsa-miR-6832-5p	#ff5723	hsa-miR-6859-5p	#5165ff
hsa-miR-6746-5p	#ca0095	hsa-miR-6765-3p	#5a0296	hsa-miR-6787-5p	#ff2192	hsa-miR-6806-5p	#00b22a	hsa-miR-6833-3p	#7467ff	hsa-miR-6860	#b500b4
hsa-miR-6747-3p	#ea0077	hsa-miR-6765-5p	#e874ff	hsa-miR-6788-3p	#ee0090	hsa-miR-6807-5p	#5f009c	hsa-miR-6833-5p	#ff0652	hsa-miR-6861-3p	#e53e00
hsa-miR-6747-5p	#0c29a6	hsa-miR-6767-5p	#ff49c2	hsa-miR-6788-5p	#f78000	hsa-miR-6808-5p	#b16bff	hsa-miR-6834-3p	#ff55e2	hsa-miR-6861-5p	#002dbc
hsa-miR-6748-3p	#ff6d2e	hsa-miR-6768-5p	#ff7024	hsa-miR-6789-3p	#af00a4	hsa-miR-6809-3p	#ff413c	hsa-miR-6835-3p	#1f69ff	hsa-miR-6862-3p	#ff4826
hsa-miR-6748-5p	#ba1d00	hsa-miR-6769a-3p	#f20081	hsa-miR-6789-5p	#ff1a89	hsa-miR-6809-5p	#ff007a	hsa-miR-6836-3p	#c900af	hsa-miR-6862-5p	#ff6903
hsa-miR-6749-3p	#4c169a	hsa-miR-6769a-5p	#ff6ff4	hsa-miR-6790-3p	#cd001a	hsa-miR-6810-3p	#e14c00	hsa-miR-6837-5p	#fb6cff	hsa-miR-6864-3p	#35c429
hsa-miR-6749-5p	#f67aff	hsa-miR-6769b-3p	#c070ff	hsa-miR-6790-5p	#c62200	hsa-miR-6812-3p	#f20049	hsa-miR-6838-5p	#ff6416	hsa-miR-6865-3p	#ff43d4
hsa-miR-675-3p	#00980d	hsa-miR-6769b-5p	#ed7000	hsa-miR-6791-5p	#d300a0	hsa-miR-6812-5p	#fb005c	hsa-miR-6839-3p	#7800a8	hsa-miR-6865-5p	#00c637
hsa-miR-6750-3p	#ff45b4	hsa-miR-6772-3p	#a56fff	hsa-miR-6792-3p	#bb00a4	hsa-miR-6813-3p	#eb5d00	hsa-miR-6839-5p	#ff65f6	hsa-miR-6866-5p	#9900b2
hsa-miR-6751-3p	#db76ff	hsa-miR-6772-5p	#d74700	hsa-miR-6792-5p	#ff147e	hsa-miR-6815-3p	#d12e00	hsa-miR-6840-3p	#59c114	hsa-miR-6867-3p	#f567ff
hsa-miR-6751-5p	#ff3c54	hsa-miR-6774-3p	#bb0a00	hsa-miR-6793-3p	#e80040	hsa-miR-6815-5p	#ff562b	hsa-miR-6841-3p	#0134c1	hsa-miR-6867-5p	#4bc31a
hsa-miR-6752-3p	#ed78ff	hsa-miR-6774-5p	#00b83a	hsa-miR-6793-5p	#e5003b	hsa-miR-6816-3p	#a700a9	hsa-miR-6845-3p	#d62900	hsa-miR-6869-5p	#9762ff
hsa-miR-6752-5p	#6f0096	hsa-miR-6775-3p	#7a009d	hsa-miR-6794-3p	#f37500	hsa-miR-6817-5p	#0154e2	hsa-miR-6845-5p	#ff6f06	hsa-miR-6870-3p	#e50021
hsa-miR-6753-3p	#ff7b24	hsa-miR-6775-5p	#a600a2	hsa-miR-6794-5p	#d13700	hsa-miR-6818-3p	#8900a6	hsa-miR-6846-3p	#ff3339	hsa-miR-6870-5p	#025cf4
hsa-miR-6753-5p	#ff59d2	hsa-miR-6776-3p	#ff2c9d	hsa-miR-6795-3p	#fa006f	hsa-miR-6818-5p	#ff3145	hsa-miR-6846-5p	#5e00a3	hsa-miR-6871-5p	#e300b3
hsa-miR-6754-3p	#ff8a1b	hsa-miR-6776-5p	#f78500	hsa-miR-6795-5p	#023ec5	hsa-miR-6819-5p	#cb000e	hsa-miR-6847-3p	#0253e6	hsa-miR-6872-3p	#a600b5
hsa-miR-6754-5p	#7572ff	hsa-miR-6777-3p	#3121a5	hsa-miR-6796-3p	#cb6eff	hsa-miR-6820-3p	#ff34b6	hsa-miR-6847-5p	#fb0048	hsa-miR-6872-5p	#0faf00
hsa-miR-6755-3p	#b90008	hsa-miR-6777-5p	#ff4d3b	hsa-miR-6796-5p	#256fff	hsa-miR-6820-5p	#3c1aa6	hsa-miR-6848-3p	#8265ff	hsa-miR-6873-3p	#7000ac
hsa-miR-6756-3p	#ff296e	hsa-miR-6778-3p	#2424a7	hsa-miR-6797-5p	#ff59dc	hsa-miR-6821-5p	#ff46cb	hsa-miR-6848-5p	#ff3735	hsa-miR-6875-3p	#0142d6
hsa-miR-6756-5p	#ff2a8a	hsa-miR-6778-5p	#f6017c	hsa-miR-6798-3p	#3faa00	hsa-miR-6823-3p	#ed009f	hsa-miR-6849-3p	#12ab00	hsa-miR-6875-5p	#9300b3
hsa-miR-6757-3p	#401c9f	hsa-miR-6779-3p	#016bf8	hsa-miR-6798-5p	#9d00a5	hsa-miR-6824-3p	#c71000	hsa-miR-6849-5p	#ff5aeb	hsa-miR-6876-5p	#8700b1
hsa-miR-6757-5p	#ce3e00	hsa-miR-6779-5p	#ff632b	hsa-miR-6799-3p	#ff30aa	hsa-miR-6824-5p	#0264f6	hsa-miR-6850-5p	#b964ff	hsa-miR-6877-3p	#ff37cb
hsa-miR-6758-3p	#c10012	hsa-miR-6780a-5p	#e66000	hsa-miR-6799-5p	#9d6cff	hsa-miR-6825-3p	#cf6aff	hsa-miR-6851-3p	#d565ff	hsa-miR-6877-5p	#f101b1
hsa-miR-6758-5p	#01ad2e	hsa-miR-6780b-5p	#d50027	hsa-miR-6800-3p	#5e6dff	hsa-miR-6825-5p	#012eb7	hsa-miR-6851-5p	#014bde	hsa-miR-6878-3p	#8c61ff
hsa-miR-6759-3p	#e50146	hsa-miR-6782-3p	#d8009c	hsa-miR-6800-5p	#b700a6	hsa-miR-6826-5p	#e90035	hsa-miR-6852-5p	#c564ff	hsa-miR-6879-3p	#4111aa
hsa-miR-6760-3p	#da5100	hsa-miR-6784-3p	#bd00a2	hsa-miR-6801-3p	#ec0097	hsa-miR-6827-3p	#e86cff	hsa-miR-6855-3p	#3d66ff	hsa-miR-6879-5p	#ff0c40
hsa-miR-6760-5p	#ff2375	hsa-miR-6784-5p	#ff1c85	hsa-miR-6801-5p	#28a400	hsa-miR-6829-5p	#de00a7	hsa-miR-6856-5p	#3eb600	hsa-miR-6880-3p	#e663ff

miRNA	HEX	miRNA	HEX	miRNA	HEX	miRNA	HEX	miRNA	HEX
hsa-miR-6880-5p	#ff06ad	hsa-miR-7107-5p	#fa4900	hsa-miR-761	#ec1400	hsa-miR-8064	#9730e2	hsa-miR-937-5p	#5238e2
hsa-miR-6881-3p	#a100b6	hsa-miR-7108-3p	#27be00	hsa-miR-762	#fe00c8	hsa-miR-8069	#f135e9	hsa-miR-939-3p	#6812c7
hsa-miR-6881-5p	#a560ff	hsa-miR-7108-5p	#705bff	hsa-miR-764	#4900b3	hsa-miR-8071	#9640f0	hsa-miR-939-5p	#431ec8
hsa-miR-6882-5p	#ff47dd	hsa-miR-7109-3p	#a459ff	hsa-miR-7641	#b552ff	hsa-miR-8072	#6b2dda	hsa-miR-942-3p	#e93aee
hsa-miR-6883-3p	#4908a8	hsa-miR-7109-5p	#3b10b1	hsa-miR-765	#f200cb	hsa-miR-8085	#ca4cfe	hsa-miR-942-5p	#eb48fa
hsa-miR-6883-5p	#6000aa	hsa-miR-711	#f90028	hsa-miR-766-3p	#ff29d9	hsa-miR-8087	#8322d5	hsa-miR-943	#724ffd
hsa-miR-6884-3p	#ff5ff8	hsa-miR-7110-3p	#ff44e6	hsa-miR-766-5p	#0249ed	hsa-miR-8088	#d328e1	hsa-miR-944	#fe1414
hsa-miR-6884-5p	#fe6000	hsa-miR-7110-5p	#105dff	hsa-miR-769-3p	#ff3feb	hsa-miR-8089	#012cd0	hsa-miR-95-3p	#272cd2
hsa-miR-6885-3p	#8100b2	hsa-miR-7111-3p	#8459ff	hsa-miR-769-5p	#e751ff	hsa-miR-8485	#d74dff	hsa-miR-9500	#ca26e0
hsa-miR-6885-5p	#eb00b6	hsa-miR-7113-3p	#565bff	hsa-miR-770-5p	#0026c7	hsa-miR-873-5p	#884bfa	hsa-miR-96-3p	#fd3cee
hsa-miR-6886-3p	#5f61ff	hsa-miR-7113-5p	#8700bc	hsa-miR-7702	#5555ff	hsa-miR-874-3p	#e22ee5	hsa-miR-96-5p	#9142f2
hsa-miR-6886-5p	#f900b2	hsa-miR-7114-3p	#ff5bff	hsa-miR-7704	#f1000a	hsa-miR-874-5p	#9c3ced	hsa-miR-98-5p	#d80ad5
hsa-miR-6887-3p	#002abd	hsa-miR-7114-5p	#00c41a	hsa-miR-7843-5p	#e500cf	hsa-miR-875-3p	#d43bef	hsa-miR-99a-5p	#1637dd
hsa-miR-6887-5p	#b200ba	hsa-miR-7150	#ac57ff	hsa-miR-7844-5p	#ff33e3	hsa-miR-876-3p	#e828e1	hsa-miR-99b-3p	#a20fd1
hsa-miR-6889-3p	#da00bb	hsa-miR-7151-3p	#ff4bef	hsa-miR-7845-5p	#003fe4	hsa-miR-876-5p	#594efa	hsa-miR-99b-5p	#611ecd
hsa-miR-6889-5p	#d000bc	hsa-miR-7152-3p	#ff2ad3	hsa-miR-7846-3p	#011abd	hsa-miR-877-5p	#fb42f3		
hsa-miR-6890-3p	#fb5600	hsa-miR-7152-5p	#ff10c6	hsa-miR-7847-3p	#6800bf	hsa-miR-885-3p	#c440f3		
hsa-miR-6890-5p	#fb0034	hsa-miR-7154-5p	#dd56ff	hsa-miR-7848-3p	#094df5	hsa-miR-885-5p	#c427e0		
hsa-miR-6891-3p	#785eff	hsa-miR-7155-3p	#ab00c5	hsa-miR-7849-3p	#433be3	hsa-miR-887-3p	#db3bef		
hsa-miR-6891-5p	#ff3326	hsa-miR-7156-3p	#c955ff	hsa-miR-7850-5p	#6d38e4	hsa-miR-888-3p	#a83cee		
hsa-miR-6892-3p	#fa5000	hsa-miR-7156-5p	#4106b1	hsa-miR-7851-3p	#b34dfe	hsa-miR-890	#7723d4		
hsa-miR-6892-5p	#df5dff	hsa-miR-7157-3p	#3013b5	hsa-miR-7854-3p	#ba2ce2	hsa-miR-892b	#a52fe3		
hsa-miR-6893-3p	#d15cff	hsa-miR-7158-3p	#ff2d1a	hsa-miR-7855-5p	#7646f3	hsa-miR-9-3p	#e215d8		
hsa-miR-6893-5p	#0148e3	hsa-miR-7159-5p	#ea0009	hsa-miR-7856-5p	#4b50fb	hsa-miR-922	#7c07c7		
hsa-miR-6894-3p	#535eff	hsa-miR-7162-3p	#0020bd	hsa-miR-7974	#271dc3	hsa-miR-92a-1-5p	#ae25dd		
hsa-miR-6894-5p	#7300b4	hsa-miR-718	#9500c3	hsa-miR-7975	#b841f3	hsa-miR-92a-3p	#8603c8		
hsa-miR-7-1-3p	#e62600	hsa-miR-744-3p	#2f59ff	hsa-miR-7976	#b500cf	hsa-miR-92b-3p	#cc0dd5		
hsa-miR-7-2-3p	#dd0002	hsa-miR-744-5p	#ff3f06	hsa-miR-7977	#3b3ee6	hsa-miR-93-3p	#a61ed8		
hsa-miR-7-5p	#f25dff	hsa-miR-7515	#f12600	hsa-miR-8052	#d748fa	hsa-miR-93-5p	#a34eff		
hsa-miR-708-5p	#0153f1	hsa-miR-758-3p	#cd00cb	hsa-miR-8054	#3826cd	hsa-miR-933	#ae13d4		
hsa-miR-7106-3p	#fc00bb	hsa-miR-758-5p	#bc00ca	hsa-miR-8060	#6a41ed	hsa-miR-936	#b536ea		
hsa-miR-7106-5p	#c700c2	hsa-miR-760	#f955ff	hsa-miR-8063	#5b24d0	hsa-miR-937-3p	#833ae9		
